# More diverse than previously thought: a novel *Hypocreaceae* symbiont from *Apterostigma* fungus-farming ants

**DOI:** 10.3897/imafungus.17.182573

**Published:** 2026-03-18

**Authors:** Mateus Oliveira da Cruz, Quimi Vidaurre Montoya, Nicole Marie Gerardo, Andre Rodrigues

**Affiliations:** 1 Department of General and Applied Microbiology, São Paulo State University (UNESP), Rio Claro, SP, Brazil Department of General and Applied Microbiology, São Paulo State University Rio Claro Brazil; 2 Department of Biology, O. Wayne Rollins Research Center, Emory University, Atlanta, GA, USA Department of Biology, O. Wayne Rollins Research Center, Emory University Atlanta United States of America

**Keywords:** ant-fungal interaction, *Apterostigma pilosum* group, Attini, *
Hypocreales
*, *
Manidigitorum
*

## Abstract

Fungi in the family *Hypocreaceae* colonize a wide range of habitats, including the nests of fungus-farming ants (*Attini*, the “attines”). Although several *Hypocreaceae* genera are known from attine ant nests, recent studies indicate an even greater, previously unrecognized diversity. In this study, we describe a new genus and five new species associated with *Apterostigma* ants. A total of 11 isolates from Brazil, Ecuador, and Panama were examined based on macro- and micromorphological characteristics, combined with a family-wide phylogenetic analysis using five molecular loci. This polyphasic approach supports the recognition of *Manidigitorum***gen. nov**. and five new species: *M. attinorum*, *M. cervicornutus*, *M. minutus*, *M. sessilis*, and *M. ramosus*. *Manidigitorum* species are distinguished from related *Hypocreaceae* by their phialidic conidiogenesis arising from an irregular-shaped basal cell resembling a hand supporting fingers. These findings broaden the known diversity of *Hypocreaceae* and provide new insights into the symbiotic relationships between fungi of this family and attine ants.

## Introduction

Due to their prolific nature, fungi in the family *Hypocreaceae* (*Ascomycota*: *Hypocreales*) colonize a wide variety of environments, including nests of fungus-farming ants (*Formicidae*: *Myrmicinae*: *Attini*: *Attina*; the “attines”) (Druzhinina et al. 2011; Kubicek et al. 2019; Montoya et al. 2019, 2023). Several genera within *Hypocreaceae* have been found strictly associated with attine ant nests (Möller 1893; Augustin et al. 2013; Meirelles et al. 2015; Montoya et al. 2019, 2021, 2023). In addition, recent systematic studies have revealed undescribed *Hypocreaceae* clades from fungus-farming ants (Montoya et al. 2021). These findings highlight that the diversity of *Hypocreaceae* fungi associated with attine ant nests is greater than previously thought.

*Hypocreaceae* fungi exhibit a wide range of morphological and ecological traits. These fungi may produce acremonium-like, gliocladium-like, pachybasium-like, or verticillium-like conidiophores with or without vesicles and form either phialoconidia or aleurioconidia. The sexual morph is unknown for many members of this group, but when present, it is typically perithecial (Rossman et al. 1999; Chaverri and Samuels 2003; Lu et al. 2004; Summerbell et al. 2018; Põldmaa et al. 2019; Du et al. 2021; Montoya et al. 2023). Although members of the *Hypocreaceae* inhabit the nests of fungus-farming ants, they are also found in a wide range of other habitats. This distribution reflects their evolution into diverse ecological roles (Druzhinina et al. 2011; Osti and Rodrigues 2018; Kubicek et al. 2019; Rodríguez et al. 2021; Chen et al. 2024). These fungi are mainly found in soil as saprobes, in symbiosis with plants as endophytes, and associated with other fungi as fungicolous fungi or mycoparasites (Põldmaa et al. 2000; Chaverri and Samuels 2013; Kubicek et al. 2019; Lakkireddy et al. 2020; Rodríguez et al. 2021; Oliveira et al. 2023). Some *Hypocreaceae* genera, such as *Escovopsis*, *Escovopsioides*, *Sympodiorosea*, and *Luteomyces*, are so far known only from associations with fungus-farming ants (Augustin et al. 2013; Montoya et al. 2021, 2023).

These attine-associated microorganisms are present both in the nests of the more derived attine ants (e.g., *Acromyrmex* and *Atta*) and in those of the more basal ones (e.g., *Apterostigma* and *Cyphomyrmex*; Gerardo et al. 2004; Masiulionis et al. 2015; Meirelles et al. 2015; Montoya et al. 2023). Nests of *Apterostigma* ants harbor several *Hypocreaceae* fungi, particularly the genera *Escovopsis*, *Escovopsioides*, *Sympodiorosea*, and the generalist fungus *Trichoderma* (Montoya et al. 2016, 2019, 2021, 2023; pers. obs.). Additionally, evidence suggests that there are also novel *Hypocreaceae* clades (referred to as clades C and D) associated with *Apterostigma* nests. These clades, phylogenetically close to *Sympodiorosea* (Montoya et al. 2021), have been found exclusively in nests of the non-leaf-cutting attine ant genus *Apterostigma* but remain undescribed. Building on these findings, in this study we aimed to describe representatives of clade D.

**Figure 1.**
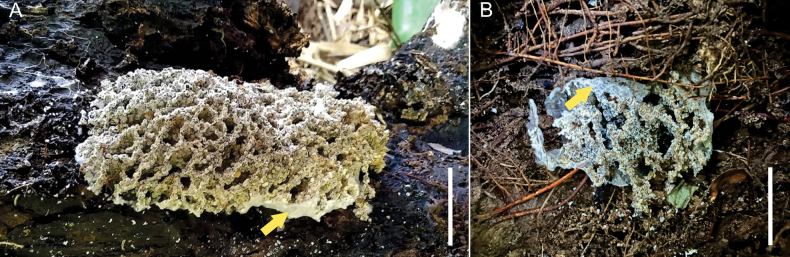
General aspect of fungus gardens of the *Apterostigma pilosum* group. **A** Fungus garden of the *Apterostigma pilosum* group found beneath a decaying log. **B** Fungus garden of the *Apterostigma pilosum* group found under leaf litter. In both colonies, remnants of the veil—the mycelial layer covering the fungus garden—are visible (arrows). Scale bar: 1 cm.

*Apterostigma* ants represent a unique lineage among the attines, as they encompass three out of the four types of fungicultures known from attines. Species in the *Apterostigma pilosum* group (Fig. 1), as well as *A. dentigerum*, *A. urichii*, and other species, differ from all other attine ants in that they cultivate fungi in the family *Pterulaceae*: *Myrmecopterula nudihortorum* and *M. velohortorum* (Leal-Dutra et al. 2020; Schultz et al. 2024). In contrast, *A. auriculatum* cultivates a *Leucocoprinae (Agaricaceae)* fungus like other lower attine genera, whereas *A. megacephala* uniquely cultivates *Leucocoprinus gongylophorus* (*Agaricaceae*), the well-known fungal cultivar of leaf-cutting attines (e.g., *Atta*, *Amoimyrmex*, and *Acromyrmex*) (Sosa-Calvo et al. 2017; Schultz et al. 2024). Thus, the description of clade D (*Hypocreaceae*) associated with fungus gardens of *Apterostigma* represents an important first step toward future studies on the ecology and evolution of the *Apterostigma* fungus-growing ant system.

Taking a multipronged approach that integrates molecular, morphological, and physiological analyses, we introduce *Manidigitorum* as a novel genus in the *Hypocreaceae*, along with five new species isolated from *Apterostigma* fungus gardens. A key feature that differentiates this clade from other *Hypocreaceae* fungi is the presence of a basal cell supporting the phialides. The discovery of this new clade increases our knowledge of the diversity and taxonomy of this family and raises new questions regarding the symbiotic relationships between these fungi and attine ants.

## Material and methods

### Fungal cultures, ant sampling, and fungal isolation

We describe 11 clade D isolates deposited in the culture collection of the Laboratory of Fungal Ecology and Systematics (LESF), São Paulo State University (UNESP), Rio Claro, Brazil. This collection houses *Hypocreaceae* fungi associated with different attine genera (e.g., *Atta*, *Acromyrmex*, *Apterostigma*, *Cyphomyrmex*, and *Mycetomoellerius*). Despite our extensive collection of *Hypocreaceae* fungi associated with different attine ant genera, isolates of *Manidigitorum* have been obtained exclusively from *Apterostigma* nests originating from tropical forests of Panama and Ecuador, as well as from the Amazon and Atlantic rainforests in Brazil. Nests of *Apterostigma* are typically found beneath or inside decaying logs, under leaf litter, in soil banks, or under rocks. Fungus gardens in which the mutualist is *Myrmecopterula velohortorum* are often covered by a layer of mycelium, referred to as a veil. In contrast, fungus gardens associated with *M. nudihortorum* lack this veil (Fig. 1). However, we did not record the presence or absence of a veil in the majority of host nests from which the *Hypocreaceae* studied here were isolated, and therefore no association can currently be inferred. Ant nest collection followed the methods described by Sosa-Calvo et al. (2015). Briefly, once the ant nests were located, we exposed the fungus gardens using flame-sterilized spoons and forceps. In some cases, scissors were used to cut roots passing through the fungus garden. Using flame-sterilized forceps, fungus gardens were transferred to small plastic containers for fungal isolation.

*Hypocreaceae* fungi were isolated using culture-dependent techniques as described in Montoya et al. (2019). Briefly, 5–7 fragments of the fungus garden (approximately 3 mm^3^ each) were plated on Potato Dextrose Agar (PDA, Neogen® Culture Media, Lansing, USA) supplemented with 150 μg mL^-1^ of chloramphenicol (Sigma-Aldrich, St. Louis, MO, USA). Three Petri dishes were used for each ant fungus garden for isolation. Plates were incubated at 25 °C in darkness and observed daily over 10 days. When mycelium of fungi grew out of the fragments, we transferred them to new PDA plates. Some isolates used in this study (LESF 870, LESF 871, LESF 875, LESF 888, LESF 889, and LESF 890) were obtained from the Mueller Lab – University of Texas, USA. For all isolates, we performed monosporic (a single conidium) culture. All isolates are preserved: (i) in sterile deionized water (Castellani 1939) and (ii) in 10% glycerol at −80 °C as conidial suspensions (cryopreserved; Jang et al. 2017) in the LESF culture collection. Holotype and ex-type cultures were deposited in the Westerdijk Fungal Biodiversity Institute (CBS), Utrecht, the Netherlands (Suppl. material 1: table S1).

### DNA extraction, PCR, and sequencing

We extracted genomic DNA using the CTAB method described in Möller et al. (1992) with modifications. Briefly, aerial mycelia from 7-day-old cultures (grown at 25 °C on PDA) were crushed using glass beads (Sigma, St. Louis, MO, USA) in lysis buffer. Subsequently, 5 μL of proteinase K (20 mg mL^-1^) was added to the suspension and incubated at 65 °C for 30 min. The organic phase of the suspension was separated by centrifugation (10,000 × *g* for 10 min) using a chloroform–isoamyl alcohol mixture (24:1). A total of 400 μL of the supernatant was collected, and the genomic DNA was precipitated using 3 M sodium acetate and 100% isopropanol. The DNA was purified through two successive washes with 70% ethanol and left at room temperature (25 °C ± 2 °C) to dry overnight. Finally, the DNA was resuspended in 30 μL of Tris–EDTA buffer and stored at −20 °C.

Five molecular loci were amplified: the internal transcribed spacer (ITS) region and the large subunit (LSU) of the ribosomal DNA (*rDNA*), the translation elongation factor 1-alpha gene (*tef*1), and the two largest subunits of RNA polymerase II (*rpb*1 and *rpb*2). The amplification reactions were carried out using primers and conditions previously published by Montoya et al. (2021, 2023). The reactions were performed in a final volume of 25 µL containing 4 µL of dNTPs (1.25 mm each); 5 µL of 5× buffer; 1 µL of BSA (1 mg mL^1^); 2 µL of MgCl_2_ (25 mm); 1 µL of each primer (10 µM); 0.2 µL of Taq polymerase (5 U µL^-1^); 2 µL of diluted genomic DNA (1:100); and 8.8 µL of sterile ultrapure water. Amplicons were purified with the Wizard SV Gel and PCR Clean-up System (Promega, Madison) following the manufacturer’s protocol. The purified amplicons were quantified using NanoDrop® (Thermo Scientific). The samples were then subjected to cycle sequencing reactions with BigDye Terminator® v.3.1 (Life Technologies), following the manufacturer’s instructions. Forward and reverse sequences were generated using an ABI 3500 (Life Technologies). Consensus sequences were assembled into contigs in Geneious v.6.0 (Kearse et al. 2012) and deposited in GenBank (accessions are provided in Suppl. material 1: table S1).

### Phylogenetic analysis

The multi-locus analysis was performed using the sequenced five loci (Suppl. material 1: table S1). We also inferred trees using a single locus to analyze conflicts between loci. The sequences were aligned separately for each locus in MAFFT v.7 (Katoh and Standley 2013). The nucleotide substitution model for each alignment was calculated in jModelTest v.2 (Darriba et al. 2012) using the Akaike Information Criterion (AIC) with 95% confidence intervals. Sequence datasets were concatenated in Geneious v.6.0 (Kearse et al. 2012). The final dataset contained a total of 68 sequences with 3817 bp in length [ITS (707 bp), LSU (601 bp), *rpb*1 (735 bp), *rpb*2 (994 bp), and *tef*1 (780 bp)]. Phylogenetic trees were reconstructed using Bayesian Inference (BI) in MrBayes v.3.2.2 (Ronquist et al. 2012) and Maximum Likelihood (ML) in RAxML v.8 (Stamatakis 2014). For the BI analysis, we carried out two separate runs (each consisting of three hot chains and one cold chain) using the GTR+I+G model for each partition independently. Two million generations of the Markov Chain Monte Carlo (MCMC) were sufficient to reach convergence (standard deviation of split frequencies < 0.01). The first 25% of trees were discarded as burn-in to generate the best BI tree. For the ML analysis, a total of 1000 independent trees were estimated using the GTR+I model. Branch support was calculated with 1000 bootstrap replicates. The final tree was visualized in FigTree v.1.4.4 (http://tree.bio.ed.ac.uk/software/figtree/) and polished in CorelDraw v.24.5.

### Morphological analysis

Macroscopic characters such as mycelium color, growth rate, and presence of soluble pigments were evaluated on three culture media: PDA, Malt Extract Agar [MEA, 30 g L^-1^ of malt extract (Neogen® Culture Media, Lansing, USA), 5 g L^-1^ of bacteriological peptone (Neogen® Culture Media, Lansing, USA), 20 g L^-1^ of glucose (Labsynth, Diadema, Brazil), and 15 g L^-1^ agar (Neogen® Culture Media, Lansing, USA)], and Cornmeal Dextrose Agar (CMD, Neogen® Culture Media, Lansing, USA). To assess the macroscopic characters, an agar fragment (ca. 5 mm in diameter × 5 mm in height) containing mycelium grown for 7 days at 25 °C on water agar was cut and placed at the center of a 90 × 15 mm Petri dish containing each test medium. The inoculated plates were incubated in darkness at 10 °C, 20 °C, 25 °C, and 30 °C, and colony diameter was measured after four and seven days using a millimeter ruler (Suppl. material 2: tables S2–S7). Media and growth conditions followed the descriptions of *Escovopsis* species (Montoya et al. 2023). The color of the colonies was described using the Kornerup and Wanscher (1978) catalog.

For the microscopic characters, we prepared slide cultures by inoculating conidia (from 7-day-old colonies at 25 °C) on fragments of PDA medium (0.5 cm in diameter) placed on the top of sterile coverslips. The coverslips were placed in Petri dishes and incubated in darkness at 25 °C. After three days, microscopic slides were prepared using a drop of lactophenol. We examined the following microscopic characters: size, color, and shape of conidiophore, basal cell, phialide, and conidia; branching pattern of the conidiophore; and number of phialides on the basal cell. The microscopic characters were photo-documented under a differential interference contrast (DIC) light microscope (DM2500 LED, Leica, Wetzlar, Germany) equipped with an image capture system (MC190HD, Leica, Wetzlar, Germany). A total of 30 measurements were made of each structure (except for some structures that were infrequent; see Suppl. material 3: tables S8–S12) using IMAGEJ v.2.9.0 (Schneider et al. 2012). Diagnostic characters distinguishing the species were used to construct the dichotomous key to species.

## Results

### Phylogenetic analysis

**Figure 2.**
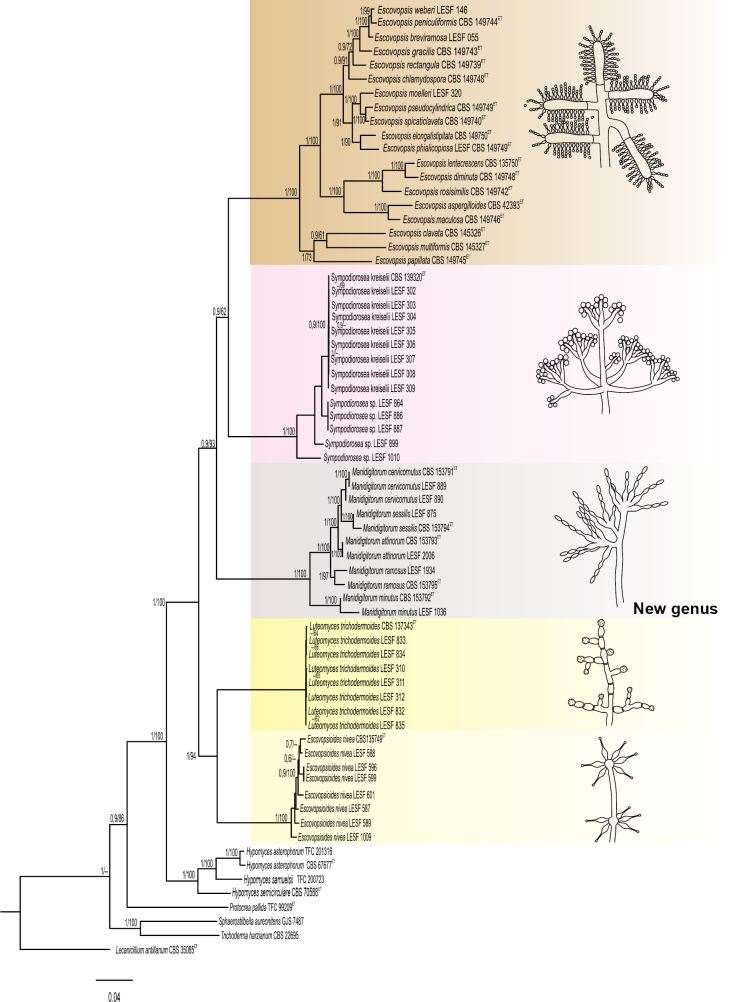
Multigene phylogeny revealing relationships among *Hypocreaceae* genera. Highlighted boxes in different colors represent the genera associated with fungus-farming ants. Representative morphology of the conidiophore of each genus is depicted to the right. The tree was inferred from 68 sequences and 3817 sites (combined dataset: ITS, LSU, *tef*1, *rpb*1, and *rpb*2). Numbers on branches indicate BI posterior probabilities > 0.69 and ML bootstrap support values > 75%. *Manidigitorum* is depicted in the gray box. *Lecanicillium antillanum*CBS 35085 is the outgroup. CBS: Westerdijk Fungal Biodiversity Institute; TFC: Tartu Fungal Culture Collection; LESF: Laboratory of Fungal Ecology and Systematics; GJS: Gary J. Samuels Collection.

**Figure 3.**
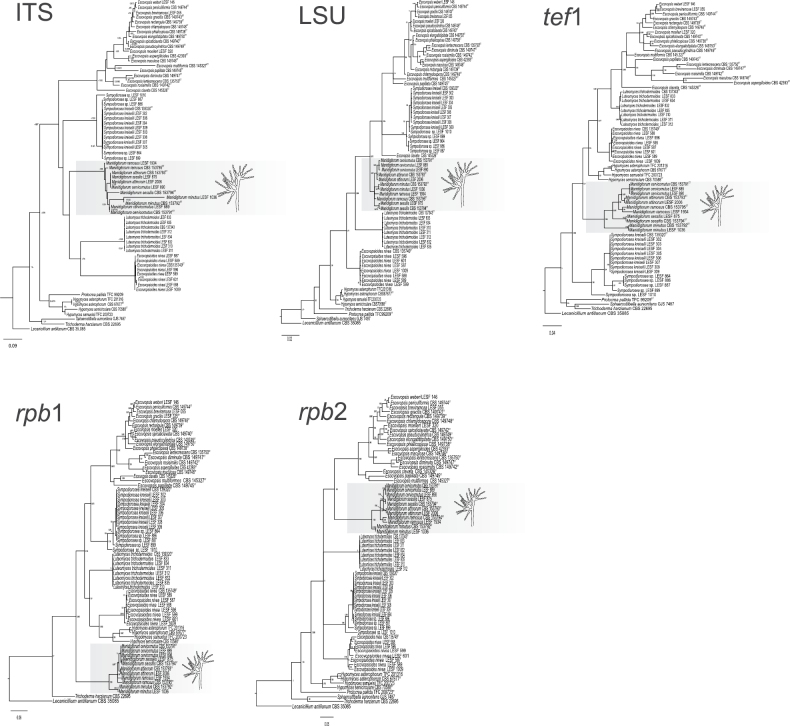
Phylogenies revealing relationships among *Hypocreaceae* genera using separate loci: ITS, LSU, *tef*1, *rpb*1, and *rpb*2. Representative morphology of the conidiophore of *Manidigitorum* is depicted on the right side. Numbers on branches indicate BI posterior probabilities > 0.69 and ML bootstrap support values > 75%. *Lecanicillium antillanum*CBS 35085 is the outgroup.

The 11 isolates formed a well-supported clade distinct from the other genera in *Hypocreaceae*. The clade was strongly supported in the combined multi-locus analysis (Fig. 2) and in most single-locus phylogenies, except for LSU, which did not provide support for the entire clade, and ITS, which showed support only under ML inference (Fig. 3). This clade is a sister lineage of *Sympodiorosea* and *Escovopsis* in the multi-locus tree [posterior probability (PP) = 0.9 and maximum likelihood bootstrap (MLB) = 93%] (Fig. 2). Among the five loci analyzed, *tef*1, *rpb*1, and *rpb*2 produced phylogenetic trees with topologies that differed from the multi-locus tree, although they still resolved clade D as monophyletic with high support (PP = 1.0 and MLB = 100%). The phylogenetic position of *Manidigitorum* varied among the five single-locus analyses, as each locus recovered different sister relationships within the *Hypocreaceae* (Fig. 3). This novel clade contains five well-supported subclades in the combined multi-locus analysis (PP = 1.0 and MLB = 97–100%, Fig. 2) and in most single-locus phylogenies (Fig. 3). Thus, we propose these clades as five new species of *Manidigitorum*: *M. attinorum*, *M. cervicornutus*, *M. minutus*, *M. ramosus*, and *M. sessilis* (Figs 2, 3).

The tree topologies differ between loci, changing the position of *Manidigitorum* species (Fig. 3). The ITS locus appears to have a poor phylogenetic signal for *Manidigitorum* species, as the tree topology is quite different from that inferred using all loci (Fig. 3). Only the topology for *M. minutus* remained preserved in the ITS. The topology of LSU, *tef*1, *rpb*1, and *rpb*2 trees differs slightly from that of the tree with all loci combined. A basal position was consistently observed for all sequences of *M. minutus* and *M. ramosus* in the trees inferred with LSU, *tef*1, *rpb*1, and *rpb*2, as well as in the combined loci tree. Among the studied loci, *tef*1 and *rpb*2 appear to exhibit better phylogenetic resolution for *Manidigitorum* species, since the topology of the trees inferred with these loci is similar to that of the multi-locus analysis (Fig. 3).

### Morphological analysis

The *Manidigitorum* isolates exhibited growth on CMD, MEA, and PDA at 20, 25, and 30 °C. No growth was observed in colonies incubated at 10 °C (Suppl. material 2: tables S2–S7). Colonies began to grow after three days of incubation. In contrast, the ex-type strains of *M. sessilis* (LESF 888) and *M. attinorum* (LESF 1034) had delayed growth at 30 °C, with visible growth only after five days. Overall, cultures grown on PDA for seven days at 25 °C provided the optimal conditions for morphological analysis, as sporulation was most abundant and colony diameter reached up to 3.5 cm. Under this culturing regime, *Manidigitorum* forms white, cottony colonies without pigmentation. The colonies of *M. attinorum* and *M. ramosus* slightly deviated from this pattern, displaying greyish-yellow and pale-yellow pigmentation, respectively, at the center of the colonies on PDA after seven days at 25 °C.

Micromorphological analyses support the phylogenetic evidence for recognizing *Manidigitorum* as a new genus within the *Hypocreaceae*. Isolates of *Manidigitorum* possess an irregularly shaped basal cell that supports the phialides, resembling a human hand with fingers. In *M. minutus* and *M. sessilis*, an additional supporting cell is present between the basal cell and the phialide. To our knowledge, no other genera in the *Hypocreaceae* exhibit these morphological features in the conidiophore.

### Taxonomy

#### Manidigitorum

Taxon classificationFungiHypocrealesHypocreaceae

M.O. Cruz, Q.V. Montoya & A. Rodrigues gen. nov.

E366A977-76B2-537B-939E-5FB07BD34773

860525

Fig. 4

##### Etymology.

“*Manidigitorum*” (manus = hand, digitus = hand fingers) in reference to the basal cell with a shape resembling a human hand supporting fingers.

**Figure 4.**
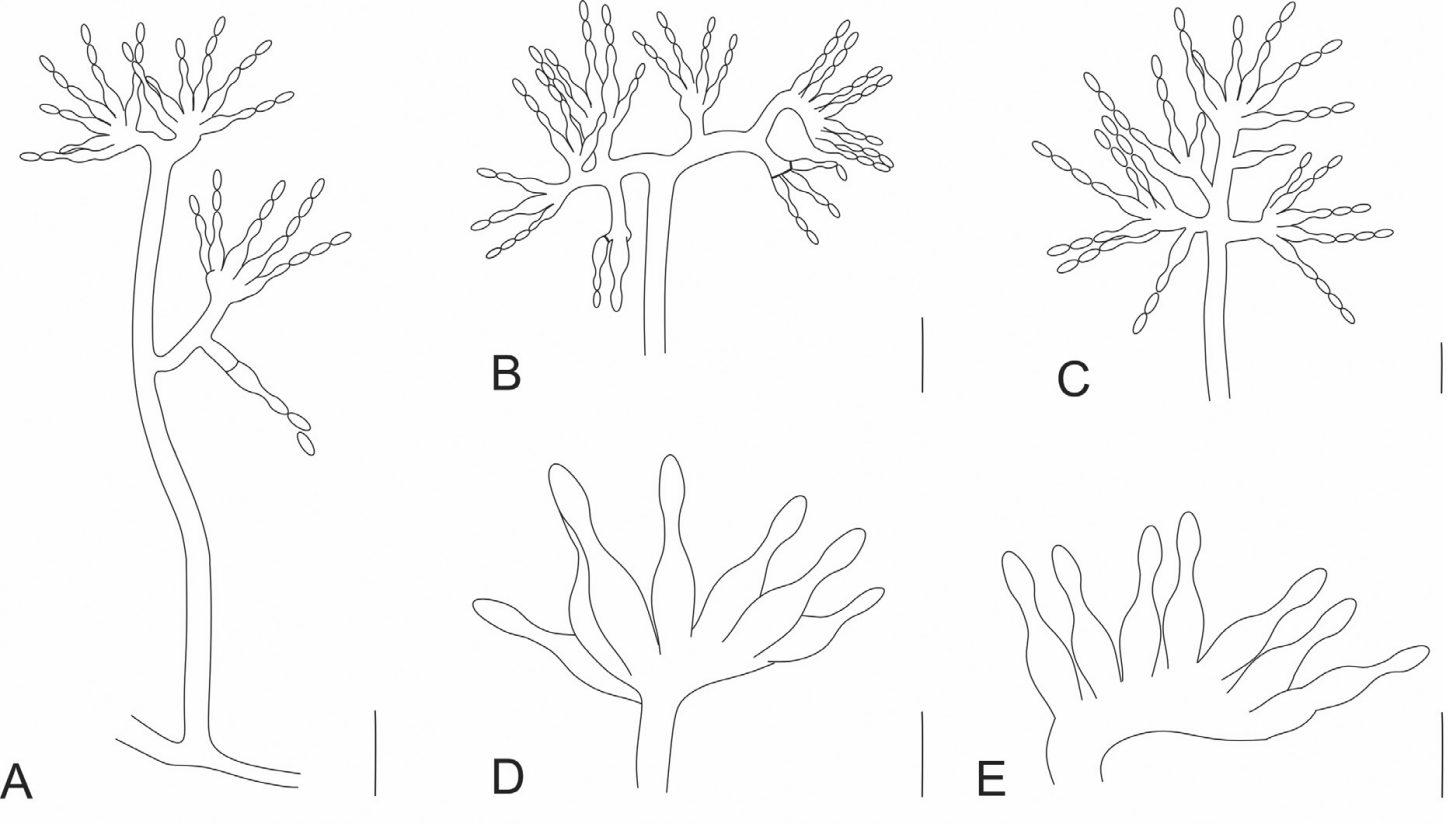
Morphology of *Manidigitorum*. **A** General morphology of the conidiophore on aerial mycelia—note a phialide with a basal septum emerging from the pedicel. **B** Conidiophore apex with bifurcated ramification. **C** Conidiophore apex without bifurcated ramification—note a phialide emerging from the conidiophore stipe (sessile phialide). **D, E** Irregular basal cell supporting phialides—in drawing **E**, there is one phialide preceded by the supporting cell. Scale bars: 50 µm (**A**); 20 µm (**B**, **C**); 10 µm (**D**, **E**).

##### Diagnosis.

Basal cells supporting phialides. These basal cells have a shape resembling a hand supporting fingers.

##### Type species.

*Manidigitorum sessilis* M.O. Cruz, Q.V. Montoya & A. Rodrigues.

##### Description.

Monophyletic group belonging to the *Hypocreaceae* with branched or unbranched conidiophores, irregularly shaped basal cells, indeterminate or vesicle-like. These cells are formed from the conidiophore stipe, conidiophore branch, or pedicel. Additional supporting cells can be observed between the basal cell and the phialides. Phialides (enteroblastic conidiogenous cells) sessile or emergent from basal cell, supporting cell, pedicel, or conidiophore branch. Conidia fusiform to oblong.

##### Notes.

*Manidigitorum* is phylogenetically placed within the *Hypocreaceae* as a sister clade of *Sympodiorosea* and *Escovopsis*. However, *Manidigitorum* fungi have white sporulation, unlike *Sympodiorosea* (pink sporulation) and *Escovopsis* (brown sporulation). In addition, *Manidigitorum* does not have sympodial conidiogenous cells, as *Sympodiorosea* does, and *Manidigitorum* does not have vesicles supporting phialides, as *Escovopsis* does.

#### Manidigitorum attinorum

Taxon classificationFungiHypocrealesHypocreaceae

M.O. Cruz, Q.V. Montoya & A. Rodrigues sp. nov.

E10EB8A0-9E0F-5850-9E7C-E9B4CBE61BA0

860526

Fig. 5

##### Etymology.

“attinorum” in reference to the subtribe *Attina* that comprises fungus-farming ants from which this fungus was isolated.

**Figure 5.**
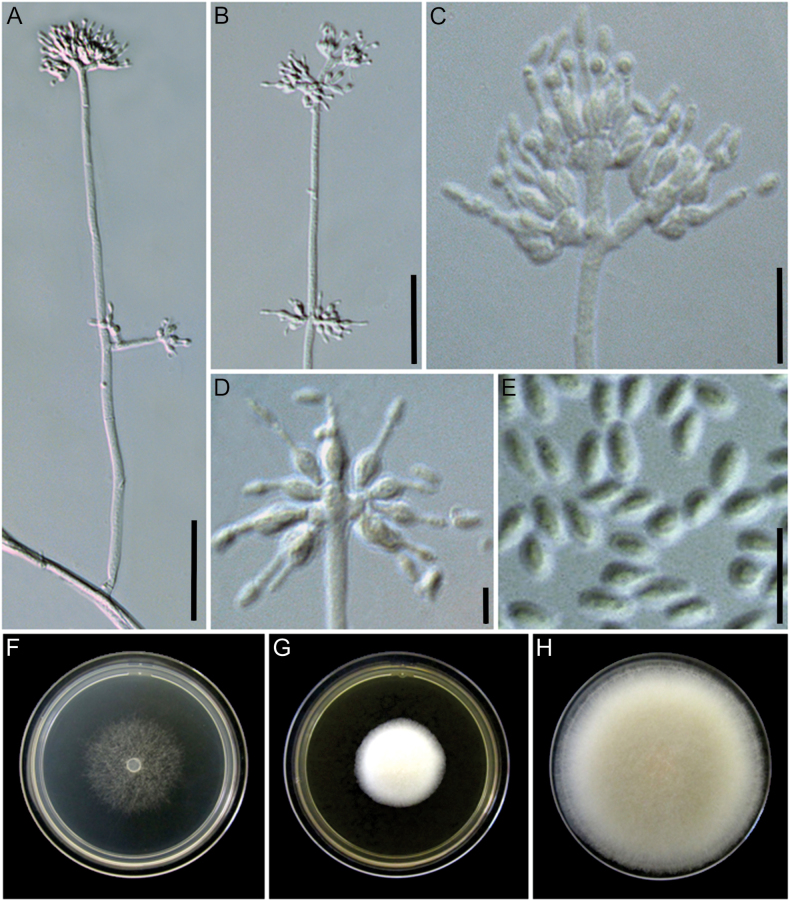
Morphological characters of *Manidigitorum attinorum* (ex-type culture CBS 153793). **A** Conidiophore from aerial hyphae. **B** Conidiophore stipe with pedicels. **C** Conidiophore tip with three basal cells. **D** Conidiophore tip arranged in a crucifix. **E** Conidia. **F–H** Culture on CMD, MEA, and PDA, respectively, after 7 days of growth at 25 °C. Scale bars: 25 µm (**A**, **B**); 10 µm (**C**, **D**); 5 µm (**E**).

##### Diagnosis.

*Manidigitorum attinorum* typically forms conidiophores with three basal cells arranged in a cruciform configuration, comprising one central basal cell and two lateral cells originating from the main axis.

##### Typus.

BRAZIL • Pará, Belterra, Floresta Nacional de Tapajós, 3°2.91'S, 54°55.68'W, on fungus garden of *Apterostigma urichii* (ant colony ID: CALD170314-03), 14 Mar 2017, Q.V. Montoya, LESF 1034 (ITS: PX225019, LSU: PX225029, *tef*1: PX633031, *rpb*1: PX663411, *rpb*2: PX663422). Holotype CBS 153793 preserved as metabolically inactive culture (ex-type culture CBS 153793).

##### Description.

Conidiophore stipe 60–284.5 × 2.5–7.5 µm, upright, alternate, or opposite along the aerial mycelium, hyaline, without a foot cell, smooth-walled, forming branches 6–35 × 2.5–4 µm; tip with up to six basal cells, frequently with three basal cells arranged in a cruciform configuration (one central basal cell and two opposite lateral cells). Pedicel 1.5–10 × 2–3.5 µm, erect, aseptate, hyaline, arising from the conidiophore stipe or branches, smooth-walled, supporting one phialide. Basal cell 2–10 × 2–6 µm, arising from the conidiophore branches or the pedicel, irregularly shaped, resembling a hand supporting fingers and rarely exhibiting a deer-horn-like appearance, hyaline, smooth-walled, supporting 1–25 phialides. Supporting cell not observed. Phialide formed on the basal cell, pedicel, or branch, rarely sessile on the conidiophore stipe, hyaline, sometimes with a basal septum, 5.5–21 µm long, lageniform, 1–3.5 × 1–5 µm at the base, 2–5.5 × 2–6 µm at the swollen section, 2.5–7.5 × 0.5–2 µm at the neck. Conidia formed in long chains, fusiform with rounded tips, 4.5–7 × 2–3 µm, hyaline, smooth-walled. Chlamydospores not observed.

Culture growth characteristics: Colonies growing at 20 °C and 25 °C on MEA, PDA, and CMD. No growth at 10 °C and 30 °C after 4 days on all media. Colony radius, after 4 days at 20 °C: 6 mm on MEA (inconspicuous growth, the colony barely grows on the inoculum), 26 mm on PDA, 13 mm on CMD; at 25 °C: 11 mm on MEA, 31 mm on PDA, 13 mm on CMD. Colony radius, after 7 days at 20 °C: 10 mm on MEA, 35 mm on PDA, 20 mm on CMD; at 25 °C: 15 mm on MEA, 35 mm on PDA, 21 mm on CMD. Colony morphology: CMD 25 °C, 7 days: colonies with loose aerial mycelium, white (1A1); MEA 25 °C, 7 days: colonies with cottony aerial mycelium, white; PDA 25 °C, 7 days: colonies with cottony aerial mycelium, greyish yellow (3B3) to white. Soluble pigments absent.

##### Distribution.

This species is found in the Amazon rainforest in Brazil.

##### Habitat.

Fungus gardens of *Apterostigma* ant nests.

##### Additional material examined.

BRAZIL • Pará, Floresta Nacional de Carajás, 6°07'57.5"S, 50°21'33.7"W, on fungus garden of *Apterostigma pilosum* group (ant colony ID: QVM241008-02), 13 Oct 2024, M.O. Cruz, LESF 2006 (ITS: PX225024, LSU: PX225030, *tef*1: PX633032, *rpb*1: PX663412, *rpb*2: PX663423).

##### Notes.

*Manidigitorum attinorum* is closely related to *M. sessilis* and *M. ramosus*. The conidiophore of *M. attinorum* is smaller than that of *M. sessilis*, and the basal cell supports more phialides. Few sessile phialides are observed in *M. attinorum*. Unlike *M. ramosus*, strains of *M. attinorum* do not have frequent branches and have smaller basal cells but support more phialides. In addition, the colony radius of *M. attinorum* on PDA and CMD at 25 °C after 4 days is greater than that of *M. sessilis* and *M. ramosus*.

#### Manidigitorum cervicornutus

Taxon classificationFungiHypocrealesHypocreaceae

M.O. Cruz, Q.V. Montoya & A. Rodrigues sp. nov.

BACDE284-0D22-5090-B3F5-5151328619BA

860527

Fig. 6

##### Etymology.

“cervicornutus” (cervus = deer, cornu = horn) in reference to the appearance of the basal cell supporting phialides.

**Figure 6.**
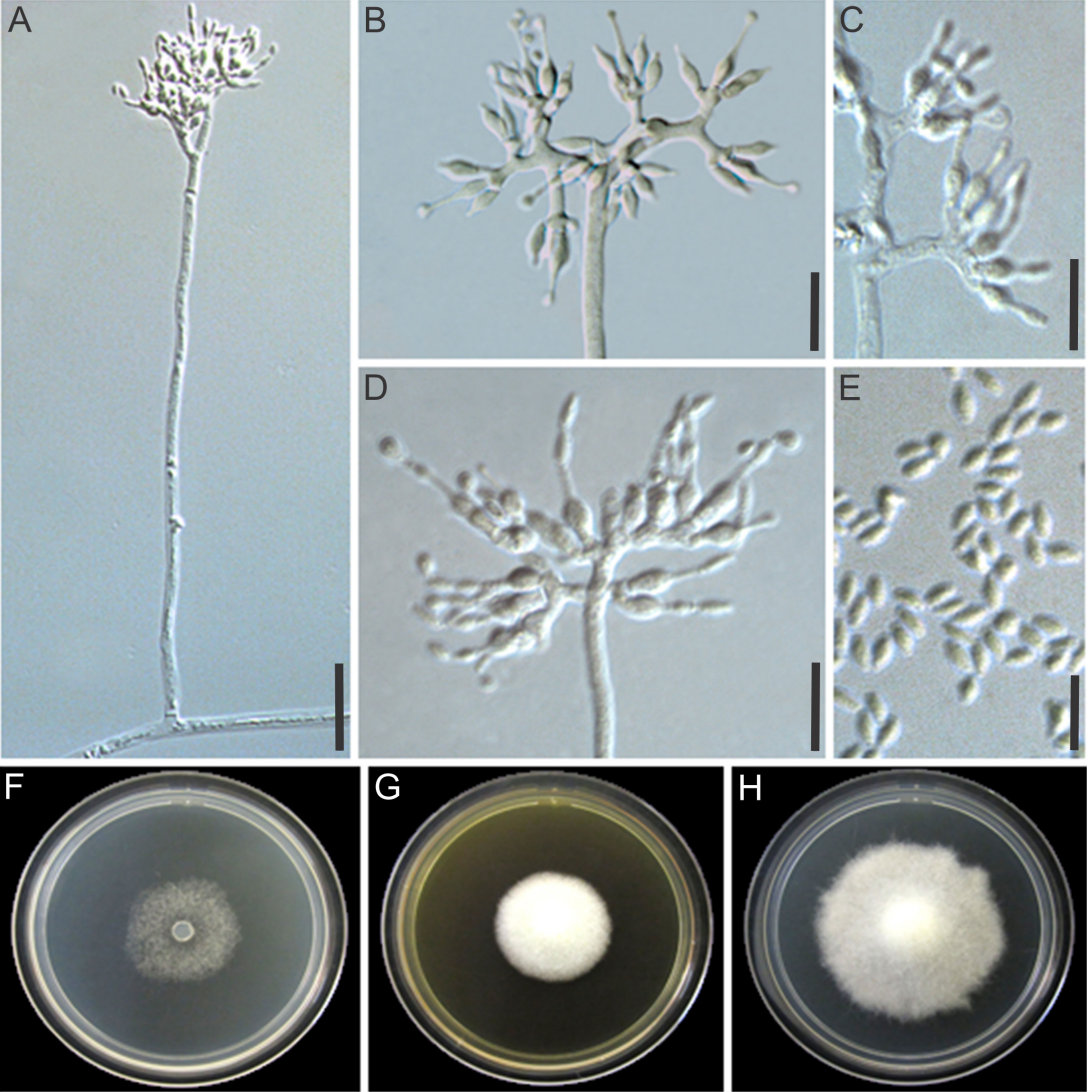
Morphological characters of *Manidigitorum cervicornutus* (ex-type culture CBS 153791). **A** Conidiophore from aerial hyphae. **B** Conidiophore tip with nine basal cells. **C** Phialides from the pedicel and from the basal cell. **D** Basal cell with irregular shape resembling a deer horn appearance. **E** Conidia. **F–H** Culture on CMD, MEA, and PDA, respectively, after 7 days of growth at 25 °C. Scale bars: 25 µm (**A**); 10 µm (**B–E**).

##### Diagnosis.

*Manidigitorum cervicornutus* frequently forms hand cells with deer horn appearance.

##### Typus.

PANAMA • Darién Province, Rancho Frio, 8°01'11.0"S, 77°43'56.5"W, on fungus garden of *Apterostigma pilosum* group (ant colony ID: UGM030106-02), U.G. Mueller, LESF 870 (ITS: PX225025, LSU: PX225026, *tef*1: PX633029, *rpb*1: PX663406, *rpb*2: PX663417). Holotype CBS 153791 preserved as metabolically inactive culture (ex-type culture CBS 153791).

##### Description.

Conidiophore stipes 80–590 × 2–5.5 µm, upright, alternate along the aerial mycelium, hyaline, without a foot cell, smooth-walled, rarely forming branches 4.5–30.5 × 2.5–3.5 µm; tip with up to nine hand-like cells. Pedicel 3.5–22.5 × 2–3.5 µm, erect, aseptate, hyaline, arising from the conidiophore stipe or tip, smooth-walled, supporting one basal cell or 1–9 phialides. Basal cell 4–14.5 × 2–6 µm, arising from the conidiophore stipe or the pedicel, hyaline, smooth-walled, irregularly shaped, resembling a hand supporting fingers or frequently exhibiting a deer-horn-like appearance, supporting 2–13 phialides. Supporting cell not observed. Phialides formed on the basal cell or pedicel, hyaline, sometimes with a basal septum, 6–17 µm long, lageniform, 0.5–4 × 1–3 µm at the base, 3.5–7.5 × 2–4 µm at the swollen section, 1–8 × 0.5–2 µm at the neck. Conidia formed in short chains, fusiform with rounded tips, 3–6 × 1.5–2.5 µm, hyaline, smooth-walled. Chlamydospores not observed.

Culture growth characteristics: Colonies growing at 20 °C, 25 °C, and 30 °C on MEA, PDA, and CMD. No growth at 10 °C after 4 days on all media. Colony radius, after 4 days at 20 °C: 8 mm on MEA (inconspicuous growth, the colony barely grows on the inoculum), 18 mm on PDA, 10 mm on CMD; at 25 °C: 10 mm on MEA, 22 mm on PDA, 13 mm on CMD; at 30 °C: 11 mm on MEA, 14 mm on PDA, 11 mm on CMD. Colony radius, after 7 days at 20 °C: 15 mm on MEA, 30 mm on PDA, 17 mm on CMD; at 25 °C: 14 mm on MEA, 29 mm on PDA, 14 mm on CMD; at 30 °C: 14 mm on MEA, 28 mm on PDA, 16 mm on CMD. Colony morphology: CMD 25°C, 7 days: colonies with loose aerial mycelium, white (1A1); MEA 25 °C, 7 days: colonies with cottony aerial mycelium, white; PDA 25 °C, 7 days: colonies with cottony aerial mycelium, white. Soluble pigments absent.

##### Distribution.

This species is found in tropical forests in Panama.

##### Habitat.

Fungus gardens of *Apterostigma* ant nests.

##### Additional material examined.

PANAMA • Colón Province, on fungus garden of *Apterostigma dentigerum* (ant colony ID: NMG020521-04), N.M. Gerardo, LESF 889 (ITS: PX225016, LSU: PX225027, *tef*1: PX633031, *rpb*1: PX663407, *rpb*2: PX663418). PANAMA, Colón Province, on fungus garden of *Apterostigma pilosum* group, U.G. Mueller, LESF 890 (ITS: PX225018, LSU: PX225028, *tef*1: PX633035, *rpb*1: PX663408, *rpb*2: PX663419).

##### Notes.

*Manidigitorum cervicornutus* is closely related to *M. sessilis*. Unlike the latter, *M. cervicornutus* has a smaller conidiophore and lacks a supporting cell and a sessile phialide. In addition, *M. cervicornutus* presents growth on PDA and CMD at 30 °C after 4 days, unlike *M. sessilis*, which does not grow at 30 °C.

#### Manidigitorum minutus

Taxon classificationFungiHypocrealesHypocreaceae

M.O. Cruz, Q.V. Montoya & A. Rodrigues sp. nov.

A0C32FD8-4759-5B8E-B842-1CAF2B2FFCB2

860528

Fig. 7

##### Etymology.

“minutus” (minutus = small) in reference to the conidiophore size.

**Figure 7.**
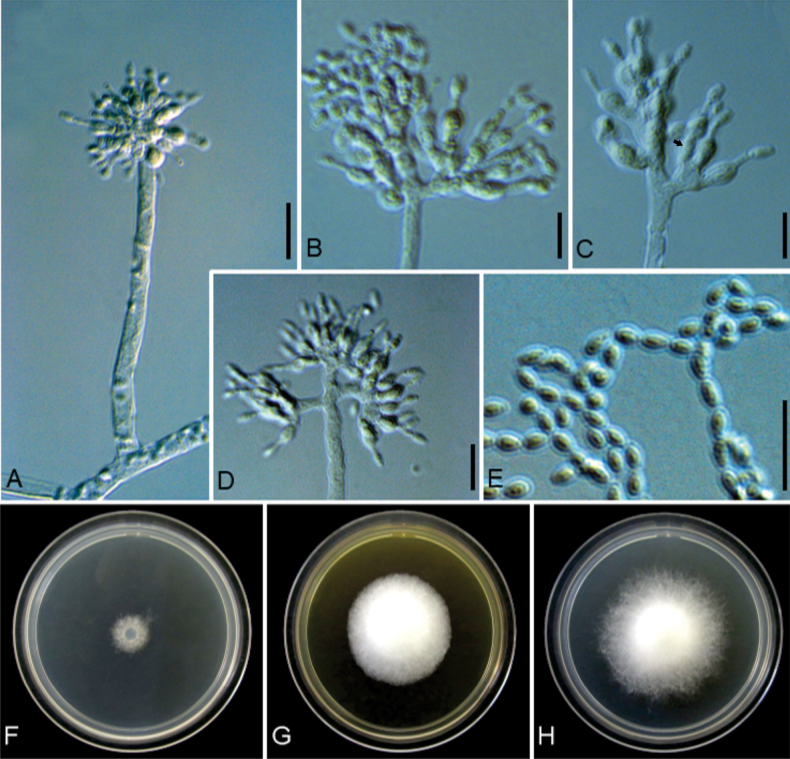
Morphological characters of *Manidigitorum minutus* (ex-type culture CBS 153792). **A** Conidiophore from aerial hyphae—the conidiophore apex supports one declined pedicel completely covered by phialides. **B** Conidiophore tip with basal cell vesicle-like supporting phialides. **C, D** Conidiophore tip with irregular and indeterminate basal cell supporting phialides (in **C**, the arrow indicates the additional supporting cell). **E** Conidia. **F–H** Culture on CMD, MEA, and PDA, respectively, after 7 days of growth at 25 °C. Scale bars: 10 µm (**A**); 8 µm (**B–C**); 6.5 µm (**D**); 10 µm (**E**).

##### Diagnosis.

*Manidigitorum minutus* forms conidiophores smaller than those of other *Manidigitorum* spp.

##### Typus.

BRAZIL • Amazonas, Novo Airão, Parque Nacional de Anavilhanas, 2°31.39'S, 60°49.53'W, on fungus garden of *Apterostigma urichii* (ant colony ID: CAR170120-04), 20 Jan 2017, Q.V. Montoya, LESF 1035 (ITS: PX225022, LSU: PX225031, *tef*1: PX633038, *rpb*1: PX663415, *rpb*2: PX663425). Holotype CBS 153792 preserved as metabolically inactive culture (ex-type culture CBS 153792).

##### Description.

Conidiophore stipe 33–250 × 3–5 µm, upright, alternate, or opposite along the aerial mycelium, hyaline, without a foot cell, smooth-walled, rarely forming branches 4–5 × 1.5–3 µm; tip with up to four basal cells. Pedicel 2–9 × 1.5–4 µm, erect or declined, aseptate, hyaline, smooth-walled, arising from the conidiophore tip, some supporting 5–15 phialides. Basal cell 4–7.5 × 2.5–5.5 µm, arising from the conidiophore stipe or pedicel, irregularly shaped, resembling a hand supporting fingers, vesicle-like or indeterminate, hyaline, smooth-walled, supporting 3–25 phialides. Supporting cell 6–10 × 1–3 µm, rare, oblong, hyaline, smooth-walled, supporting one phialide. Phialides formed on the basal cell or pedicel, rarely sessile, hyaline, sometimes with a basal septum, 6.1–12.2 µm long, lageniform, 1–2.5 × 0.5–3 µm at the base, 3–6.5 × 1.5–4 µm at the swollen section, 1.5–7.5 × 0.5–2.5 µm at the neck. Conidia formed in long chains, fusiform with rounded tips, 2–4 × 1.5–2.5 µm, hyaline, smooth-walled. Chlamydospores not observed.

Culture growth characteristics: Colonies growing at 20 °C, 25 °C, and 30 °C. No growth at 10 °C after 4 days on all media. Colony radius, after 4 days at 20 °C: 4 mm on MEA, 4 mm on PDA (inconspicuous growth, the colony barely grows on the inoculum), 12 mm on CMD; at 25 °C: 8 mm on MEA, 6 mm on PDA, 5 mm on CMD (inconspicuous growth); at 30 °C: 8 mm on MEA (inconspicuous growth), 14 mm on PDA, 7 mm on CMD (inconspicuous growth). Colony radius, after 7 days at 20 °C: 8 mm on MEA (inconspicuous growth), 15 mm on PDA, 3 mm on CMD (inconspicuous growth); at 25 °C: 1.9 mm on MEA, 22 mm on PDA, 7 mm on CMD; at 30 °C: 9 mm on MEA (the colony barely grows on the inoculum), 26 mm on PDA, 13 mm on CMD. Colony morphology: CMD 25 °C, 7 days: colonies with loose aerial mycelium, white (1A1); MEA 25 °C, 7 days: colonies with cottony aerial mycelium, white; PDA 25 °C, 7 days: colonies with cottony aerial mycelium, white. Soluble pigments absent.

##### Distribution.

This species is found in the Amazon rainforest in Brazil.

##### Habitats.

Fungus gardens of *Apterostigma* ant nests.

##### Additional material examined.

BRAZIL • Amazonas, Novo Airão, Parque Nacional de Anavilhanas, on fungus garden of *Apterostigma pilosum* group (ant colony ID: CC170119-03), 19 Jan 2017, Q.V. Montoya, LESF 1036 (ITS: PX225023, LSU: PX225032, *tef*1: PX633039, *rpb*1: PX663416, *rpb*2: PX663426).

##### Notes.

*Manidigitorum minutus* is closely related to *M. ramosus*. However, unlike *M. ramosus*, the basal cells of *M. minutus* can be vesicle-like and support a greater number of phialides. No supporting cell was observed in *M. minutus*, as seen in *M. ramosus*. Additionally, the mycelial growth of *M. minutus* on PDA (after 4 and 7 days at 20, 25, and 30 °C) is slower compared to that of *M. ramosus*.

#### Manidigitorum ramosus

Taxon classificationFungiHypocrealesHypocreaceae

M.O. Cruz, Q.V. Montoya & A. Rodrigues sp. nov.

DE7054DE-619B-5457-8C6E-C7D5D3394CB2

860529

Fig. 8

##### Etymology.

“ramosus” (ramosus = branched) in reference to the frequently branched conidiophores.

**Figure 8.**
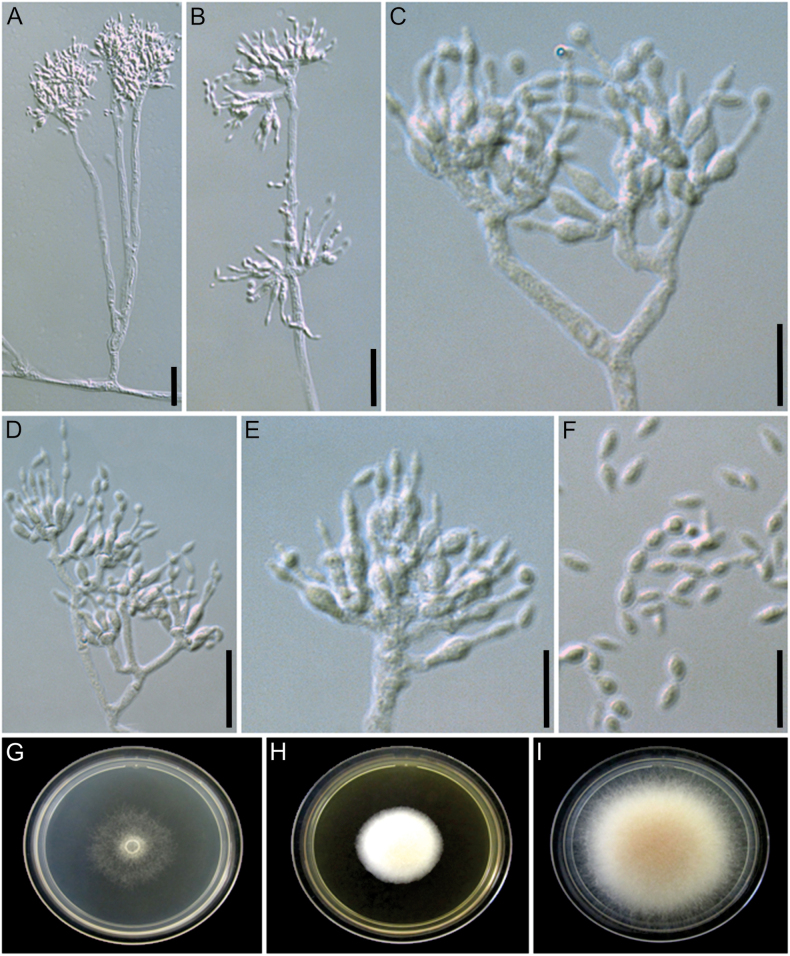
Morphological characters of *Manidigitorum ramosus* (ex-type culture CBS 153795). **A** Conidiophore from aerial hyphae. **B** Conidiophore tip showing phialides arising from the basal cell as well as from the pedicel. **C, D** Conidiophore tip with bifurcation. **E** Conidiophore tip with sessile phialide. **F** Conidia. **G–I** Culture on CMD, MEA, and PDA, respectively, after 7 days of growth at 25 °C. Scale bars: 25 µm (**A**, **B**, **F**); 10 µm (**C**, **E**); 20 µm (**D**).

##### Diagnosis.

*Manidigitorum ramosus* forms conidiophores with frequent branches.

##### Typus.

ECUADOR • eastern, Tiputini Biodiversity Station, on fungus garden of *Apterostigma cf. dentigerum* (ant colony ID: AGH030609-03), 06 Sep 2003, Anna Himler, LESF 871 (ITS: PX225020, LSU: PX225034, *tef*1: PX633033, *rpb*1: PX663414, *rpb*2: PX663424). Holotype CBS 153795 preserved as metabolically inactive culture (ex-type culture CBS 153795).

##### Description.

Conidiophore stipe 54–858 × 2–6.5 µm, upright, alternate, or opposite along the aerial mycelium, hyaline, without a foot cell, smooth-walled, always forming branches 4.5–65.5 × 2.5–6.5 µm; tip usually bifurcate, with up to seven basal cells. Pedicel 2.5–21.5 × 2–6 µm, erect, aseptate, hyaline, arising from the conidiophore stipe or branches, smooth-walled, supporting 1–5 phialides. Basal cell 2–20 × 2.5–10 µm, arising from the conidiophore branches or the pedicel, irregularly shaped, resembling a hand supporting fingers, hyaline, smooth-walled, supporting 1–9 phialides. Supporting cell not observed. Phialides formed on the basal cell, pedicel, or branch, rarely sessile on the conidiophore stipe, hyaline, sometimes with a basal septum, 6–40 µm long, lageniform, 1–9 × 1–8 µm at the base, 1.5–15 × 1.5–8 µm at the swollen section, 1–20.5 × 0.6–2.5 µm at the neck. Conidia formed in long chains, fusiform with rounded tips, 3.5–7.5 × 1.5–3 µm, hyaline, smooth-walled. Chlamydospores not observed.

Culture growth characteristics: Colonies growing at 20 °C, 25 °C, and 30 °C on MEA, PDA, and CMD. No growth at 10 °C after 4 days on all media. Colony radius, after 4 days at 20 °C: 6 mm on MEA (inconspicuous growth, the colony barely grows on the inoculum), 17 mm on PDA, 14 mm on CMD; at 25 °C: 11 mm on MEA, 22 mm on PDA, 9 mm on CMD (inconspicuous growth); at 30 °C: 11 mm on MEA, 23 mm on PDA, 10 mm on CMD. Colony radius, after 7 days at 20 °C: 10 mm on MEA, 34 mm on PDA, 5 mm on CMD; at 25 °C: 17 mm on MEA, 35 mm on PDA, 15 mm on CMD; at 30 °C: 17 mm on MEA, 34 mm on PDA, 15 mm on CMD. Colony morphology: CMD 25 °C, 7 days: colonies with loose aerial mycelium, white (1A1); MEA 25 °C, 7 days: colonies with cottony aerial mycelium, white; PDA 25 °C, 7 days: colonies with cottony aerial mycelium, white to pale yellow (4A3). Soluble pigments absent.

##### Distribution.

The species is found in tropical forests in eastern Ecuadorian Amazon and Atlantic rainforest in Brazil.

##### Habitat.

Fungus gardens of *Apterostigma* ant nests.

##### Additional material examined.

BRAZIL • São Paulo, Ribeirão Grande, Parque Estadual Intervales, 24°16.90'S, 48°24.68'W, on fungus garden of *Apterostigma* sp. (ant colony ID: DSA240510-01), 20 May 2024, M.O. Cruz, LESF 1934 (ITS: PX225015, LSU: PX225033, *tef*1: PX633034, *rpb*1: PX663413, *rpb*2: PX663427).

##### Notes.

*Manidigitorum ramosus* is closely related to *M. attinorum* and *M. minutus*. However, unlike *M. attinorum*, the basal cells in *M. ramosus* support fewer phialides. In addition, *M. ramosus* lacks a supporting cell, in contrast to *M. minutus*. The basal cells of *M. ramosus* are larger than those of *M. minutus*, and its conidiophores are larger than those of both species. The mycelial growth of *M. ramosus* on CMD (from 7-day-old cultures at 20 °C) and on PDA (from 4-day-old cultures at 20 °C) is slower than that of *M. attinorum* under the same conditions. Conversely, the mycelial growth of *M. ramosus* on PDA (from 4- and 7-day-old cultures at 20, 25, and 30 °C) is faster than that of *M. minutus*.

#### Manidigitorum sessilis

Taxon classificationFungiHypocrealesHypocreaceae

M.O. Cruz, Q.V. Montoya & A. Rodrigues sp. nov.

0AEB05D0-A14D-530A-AC11-D2D92711D33A

860530

Fig. 9

##### Etymology.

“sessilis” (sessilis = sessile) in reference to the sessile phialides.

**Figure 9.**
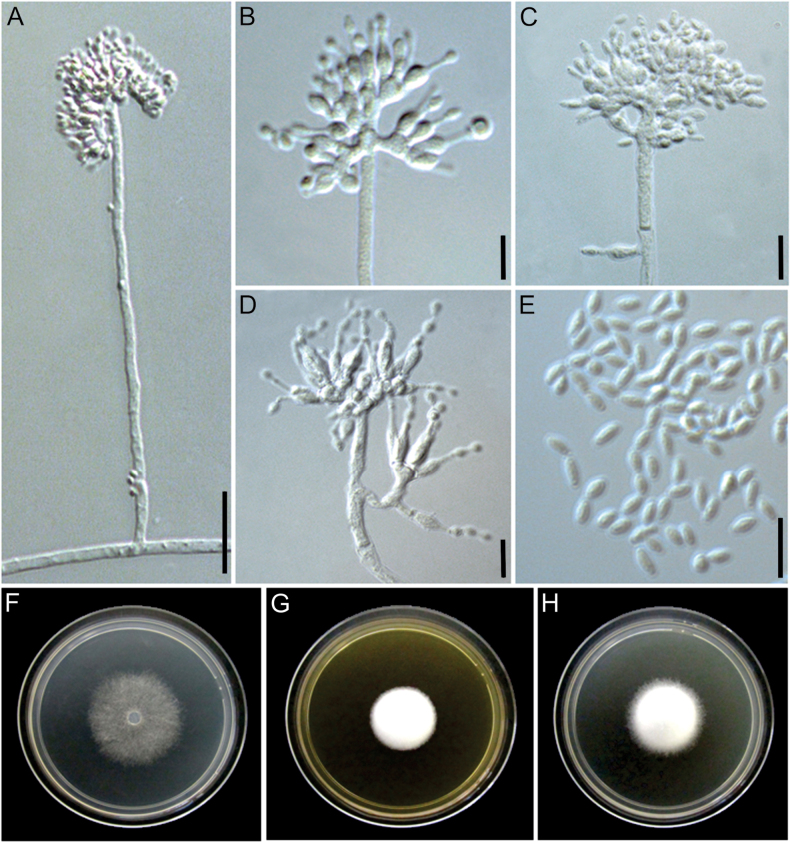
Morphological characters of *Manidigitorum sessilis* (ex-type culture CBS 153794). **A** Conidiophore with sessile phialides along the stipe. **B** Aspect of conidiophore along aerial mycelia. **C** Conidiophore apex with basal cells formed from pedicels and branches. **D** Sessile phialides. **E** Conidia. **F–H** Culture on CMD, MEA, and PDA, respectively, after 7 days of growth at 25 °C. Scale bars: 50 µm (**A**, **B**); 10 µm (**C**); 10 µm (**D**); 8 µm (**E**).

##### Diagnosis.

*Manidigitorum sessilis* forms several sessile phialides along the conidiophore.

##### Typus.

PANAMA • Bocas del Toro Province, on fungus garden of *Apterostigma pilosum* group, 21 May 2002, U.G. Mueller, LESF 888 (ITS: PX225021, LSU: PX225036, *tef*1: PX633037, *rpb*1: PX663410, *rpb*2: PX663421). Holotype CBS 153794 preserved as metabolically inactive culture (ex-type culture CBS 153794).

##### Description.

Conidiophore stipe 37–674 × 3–6.5 µm, upright, alternate, or opposite along the aerial mycelium, rarely forming branches 30–35.5 × 2.5–3.5 µm, hyaline, without a foot cell, smooth-walled; tip with up to five basal cells. Pedicel 0.5–15.5 × 0.5–6.0 µm, erect, aseptate, hyaline, arising from the conidiophore, smooth-walled, supporting one basal cell or 1–5 phialides. Basal cell 3.5–10.5 × 2.5–5 µm, arising from the conidiophore or the pedicel, irregularly shaped, resembling a hand supporting fingers, hyaline, smooth-walled, supporting 1–13 phialides. Supporting cell 7.5–13 × 2–6 µm, oblong, hyaline, smooth-walled, supporting one phialide. Phialides formed on the basal cell, pedicel, or supporting cell, frequently sessile, hyaline, sometimes with a basal septum, 7.5–15.5 µm long, lageniform, 1–5 × 1–6 µm at the base, 3.5–9 × 2–4 µm at the swollen section, 2–6.5 × 1–3 µm at the neck. Conidia formed in long chains, fusiform to oblong, 2.5–4 × 1–2.5 µm, hyaline, smooth-walled. Chlamydospores not observed.

Culture growth characteristics: Colonies growing 20 °C and 25 °C on MEA, PDA, and CMD. No growth at 10 °C and 30 °C with 4 days on all media analyzed. Colony radius, after 4 days at 20 °C: 3 mm on MEA (inconspicuous growth, the colony barely grows on the inoculum), 6 mm on PDA (inconspicuous growth), 13 mm on CMD; at 25 °C: 5 mm on MEA, 9 mm on PDA, 10 mm on CMD (inconspicuous growth). Colony radius, after 7 days at 20 °C: 9 mm (inconspicuous growth), 15 mm on PDA, 16 mm on CMD; at 25 °C: 11 mm on MEA, 15 mm on PDA, 19 mm on CMD. Colony morphology: CMD 25 °C, 7 days: colonies with loose aerial mycelium, white (1A1); MEA 25 °C, 7 days: colonies with cottony aerial mycelium, white; PDA 25 °C, 7 days: colonies with cottony aerial mycelium, white. Soluble pigments absent.

##### Distribution.

This species is found in tropical forests in Panama and the Atlantic rainforest in Brazil.

##### Habitat.

Fungus gardens of *Apterostigma* ant nests.

##### Additional material examined.

PANAMA • Bocas del Toro Province, on fungus garden of *Apterostigma cf. dentigerum* (ant colony ID: UGM020602-07), 02 Jun 2002, U.G. Mueller, LESF 875 (ITS: PX225017, LSU: PX225035, *tef*1: PX633036, *rpb*1: PX663409, *rpb*2: PX663420).

##### Notes.

*Manidigitorum sessilis* is closely related to *M. attinorum* and *M. cervicornutus*. However, unlike both species, strains of *M. sessilis* form supporting cells. In addition, the mycelial growth of *M. sessilis* on PDA (from 4- and 7-d-old cultures at 20, 25, and 30 °C) is slower than that of *M. attinorum*. Unlike *M. cervicornutus*, strains of *M. sessilis* show no mycelial growth on PDA, MEA, or CMD at 30 °C after 4 days.

### Dichotomous key to species of *Manidigitorum*

**Table:** 

1	Conidiophore stipes not exceeding 250 µm in length; basal cell vesicle-like	***M. minutus***
–	Conidiophore stipes exceeding 250 µm in length; basal cell not vesicle-like	**2**
2	Sessile phialides frequent along the conidiophore	***M. sessilis***
–	Sessile phialides rare along the conidiophore	**3**
3	Conidiophores frequently bifurcating at the tips	***M. ramosus***
–	Conidiophores rarely bifurcating at the tips	4
4	Conidiophores frequently with three basal cells arranged in a cruciform configuration (one central basal cell and two opposite lateral cells); basal cells rarely exhibiting a deer-horn-like appearance	***M. attinorum***
–	Conidiophore rarely with three basal cells arranged in a cruciform configuration; basal cells frequently exhibiting a deer-horn-like appearance	***M. cervicornutus***

## Discussion

Species of *Hypocreaceae* display diverse morphologies and lifestyles, and some *Hypocreaceae* fungi regularly inhabit the fungus-farming ant system. The main genera found in this symbiosis are *Escovopsis*, *Escovopsioides*, *Luteomyces*, *Sympodiorosea*, and *Trichoderma* (Montoya et al. 2016, 2021, and references therein). The latter is a generalist fungus, exhibiting a variety of habitats and lifestyles, from saprobe to parasite (Atanasova et al. 2013; Chaverri and Samuels 2013; Oliveira et al. 2023). In contrast, the other genera appear to have a lifestyle restricted to symbiosis with attine ants, as they have been found exclusively in this system (Montoya et al. 2021). Different studies have described new *Hypocreaceae* clades associated with attine ants (Augustin et al. 2013; Meirelles et al. 2015; Montoya et al. 2016; Marfetán et al. 2018; Montoya et al. 2019, 2021, 2023). Despite these advances, several clades of *Hypocreaceae* associated with attine ants remain undescribed. Overall, the diversity of *Hypocreaceae* fungi within this symbiotic system appears to be higher than previously thought (Montoya et al. 2021).

*Hypocreaceae* comprises approximately 21 genera (Wijayawardene et al. 2022). Fungi in this family commonly form phialidic conidiogenous cells arranged in verticils (e.g., *Trichoderma* – Zheng et al. 2021) or on vesicles (e.g., *Escovopsis* – Montoya et al. 2023) and, less frequently, sympodial (e.g., *Sympodiorosea* – Meirelles et al. 2015) and poorly differentiated conidiogenous cells (e.g., *Luteomyces* – Masiulionis et al. 2015). Furthermore, in addition to phialoconidia, *Hypocreaceae* fungi can also produce aleurioconidia (e.g., *Escovopsioides*, *Mycogone*, and *Sepedonium* – Sahr 1999; Rossman et al. 1999; Augustin et al. 2013; Põldmaa et al. 2019; Binimelis-Salazar et al. 2021).

Species of *Manidigitorum* are morphologically different from other *Hypocreaceae* fungi. Notably, among the genera in this family, only *Manidigitorum* is known to form irregular hand-shaped basal cells supporting phialides. Future research may clarify the evolutionary factors that led *Manidigitorum* species to be the only known fungi in the *Hypocreaceae* to have this unusual form. *Manidigitorum* is also distinct from some species of *Hypocreaceae* that form sexual perithecial ascomata (Rossman et al. 1999; Johnston et al. 2007) or, in some cases, both sexual and asexual structures (Chaverri and Samuels 2003; Chaverri et al. 2003; Lu et al. 2004). In the case of *Manidigitorum*, sexual structures were not observed. This is not, however, unique to *Manidigitorum*, as no sexual structures have been observed for other genera, including *Escovopsioides*, *Escovopsis*, *Kiflimonium*, *Luteomyces*, *Mycogone*, *Sepedonium*, *Sporophagomyces*, *Stephanoma*, and *Sympodiorosea* (Põldmaa et al. 2019; Augustin et al. 2013; Summerbell et al. 2018; Binimelis-Salazar et al. 2021; Du et al. 2021; Montoya et al. 2021, 2023).

The macroscopic characteristics of *Manidigitorum* also differ from those of its related genera (*Escovopsis*, *Escovopsioides*, *Luteomyces*, and *Sympodiorosea*). *Manidigitorum* growth is faster than the basal clades of *Escovopsis* (e.g., *E. clavata* and *E. multiformis*) and slower than the most derived *Escovopsis* clades (e.g., *E. rectangula* and *E. weberi*) (Montoya et al. 2023). *Manidigitorum* species grow slower than *Luteomyces trichodermoides* (Masiulionis et al. 2015) but faster than *Escovopsioides nivea* (Augustin et al. 2013) and similarly to *Sympodiorosea kreiselii* (approx. 2–3 cm) (Meirelles et al. 2015). The colony color is also a distinctive characteristic among these related genera. Cultures of *Manidigitorum* are white, like those of *Escovopsioides*, and differ from *Escovopsis* (brown culture), *Luteomyces* (yellow culture), and *Sympodiorosea* (pink culture). Based on these macroscopic characteristics, *Manidigitorum* members have already been treated as distinct from these other genera and termed “white *Escovopsis*” (Currie et al. 2003; Gerardo et al. 2004) before the reassessment of *Escovopsis*, which proposed splitting the genus into multiple genera (Montoya et al. 2021). In the reassessment of *Escovopsis*, isolates of “white *Escovopsis*” formed one clade, named ‘clade D’ (Montoya et al. 2021). Clade D contains the newly described genus *Manidigitorum*.

*Manidigitorum* species are morphologically similar to each other. The main features that distinguish the species are conidiophore length and branching pattern, sessile phialide frequency, and shape of the basal cell. Future investigations may clarify the evolutionary factors that resulted in the pronounced morphological similarity among these species. Among the phylogenetically related groups of *Manidigitorum* that are specific to attine nests, interspecific comparisons can only be made with *Escovopsis* species. This is because *Escovopsioides* (Augustin et al. 2013), *Luteomyces*, and *Sympodiorosea* are currently monotypic (Montoya et al. 2021). Species of *Escovopsis* are morphologically differentiated based on macroscopic and physiological features observed on a range of media. These characters were used in the dichotomous key for species identification (Montoya et al. 2023). Moreover, *Escovopsis* subclades can be morphologically distinguished by the shape of the vesicle, which appears to represent adaptations of the species over evolutionary time within the attine symbiosis (Montoya et al. 2025). Similarly, future studies may investigate whether *Manidigitorum* exhibits morphological adaptive traits related to its association with *Apterostigma* ants and their fungal cultivars.

The ant host range of *Manidigitorum* offers intriguing insights into the ecology of *Hypocreaceae* fungi associated with attine ants. Whereas *Escovopsis*, *Escovopsioides*, *Luteomyces*, and *Sympodiorosea* occur in nests of multiple attine genera (Meirelles et al. 2015; Masiulionis et al. 2015; Marfetán et al. 2018; Montoya et al. 2019, 2021, 2023, 2025), if *Manidigitorum* indeed exhibits a single attine ant genus association, this would represent a novel pattern within the attine symbiosis. To date, *Manidigitorum* has been isolated from fungus gardens of members of the *A. pilosum* group, as well as *A. dentigerum* and *A. urichii*, all of which cultivate *Myrmecopterula (Pterulaceae)* (Schultz et al. 2024). On the other hand, *Manidigitorum* has not been found in association with *A. megacephala* (which cultivates *L. gongylophorus*) and *A. auriculatum* (which cultivates *Leucocoprinae* fungi). If *Manidigitorum* is indeed restricted to *Apterostigma* ants that cultivate *Pterulaceae*, this association may reflect an evolutionary relationship between *Manidigitorum* and *Myrmecopterula*. However, because we did not assess the presence or absence of *Manidigitorum* across other fungiculture systems, we cannot conclude that it is exclusively associated with *Apterostigma* gardens. This limitation restricts any inference about the possible evolution of the *Manidigitorum–Myrmecopterula* association. Future studies, ideally based on extensive sampling, should investigate the distribution of *Manidigitorum* across the different fungiculture systems described for attine ants (Schultz et al. 2024) as well as clarify the evolutionary aspect of the genus.

The ecological role of *Manidigitorum* species in *Apterostigma* nests remains unknown. Given that symbioses span neutral, beneficial, and negative interactions (Moran 2006), any of these association types may characterize the relationship between *Manidigitorum* and *Myrmecopterula* or other members of the *Apterostigma* fungus-farming system (e.g., worker ants, actinobacteria, and *Escovopsis*) (Ješovnik et al. 2016; Goldstein and Klassen 2020; Montoya et al. 2023). The related genera of *Manidigitorum* have different lifestyles in the attine ant system. Some species of *Escovopsis*, such as *E. weberi*, are able to parasitize the attine cultivars and can rapidly overgrow nests in laboratory assays of virulence (Currie 2001; Reynolds and Currie 2004). Other *Escovopsis* species, however, may be opportunists living in attine ant nests (Jiménez-Gómez et al. 2021). Similarly, species of *Sympodiorosea* and *Escovopsioides* can be antagonists of the fungi cultivated by *Cyphomyrmex* (Gerardo et al. 2004), *Mycetophylax* (Custódio and Rodrigues 2019), and leaf-cutting ants (Pietrobon et al. 2022), but much of this antagonism appears to be minimized in healthy gardens, where ants appear to be able to carefully manage the fungus garden and the associated microbial community. Given that closely related clades share evolutionary histories that may influence their ecological traits, the interaction of *Manidigitorum* within the fungus-farming ant system may resemble that of its related genera. Future experimental research will be needed to determine the ecological role (e.g., mutualism, antagonism, or parasitism) of *Manidigitorum*.

## Conclusion

Different genera of *Hypocreaceae* fungi are specific to attine ants and share a common evolutionary history. New clades of fungi living in association with these ants have been inferred within the *Hypocreaceae* phylogenetic tree. Here, by integrating classical and multi-locus taxonomic approaches, we demonstrate that one of these clades displays both genetic and morphological differences from all previously described genera in the family. Accordingly, we propose *Manidigitorum* as a new genus, comprising five species described herein. The occurrence patterns of these new clades suggest ecologically distinctive features that deserve further investigation, as they may provide new insights into the ecological and evolutionary dynamics of the fungus-farming ant symbiosis.

## Supplementary Material

XML Treatment for Manidigitorum

XML Treatment for Manidigitorum attinorum

XML Treatment for Manidigitorum cervicornutus

XML Treatment for Manidigitorum minutus

XML Treatment for Manidigitorum ramosus

XML Treatment for Manidigitorum sessilis

## Additional information

### Use of AI

AI was used for correcting English grammar and style only.

### Adherence to national and international regulations

All the fungal strains used in this study have been legally obtained, respecting the Convention on Biological Diversity (Rio Convention).

### Author ORCIDs

Mateus Oliveira da Cruz https://orcid.org/0000-0002-5082-0117

Quimi Vidaurre Montoya https://orcid.org/0000-0002-3285-5981

Nicole Marie Gerardo https://orcid.org/0000-0002-3185-6616

Andre Rodrigues https://orcid.org/0000-0002-4164-9362

### Data availability

All of the data that support the findings of this study are available in the main text and in the supplementary materials.

## Supplementary materials

Supplementary material 1 Metadata of strains used in the phylogenetic analyses Data type docx Explanation note table S1. https://binary.pensoft.net/file/1564483This dataset is made available under the Open Database License (http://opendatacommons.org/licenses/odbl/1.0/). The Open Database License (ODbL) is a license agreement intended to allow users to freely share, modify, and use this Dataset while maintaining this same freedom for others, provided that the original source and author(s) are credited. Mateus Oliveira da Cruz, Quimi Vidaurre Montoya, Nicole Marie Gerardo, Andre Rodrigues

Supplementary material 2 Colony growth diameter of *Manidigitorum* isolates under varying culture conditions Data type xlsx Explanation note tables S2–S7. https://binary.pensoft.net/file/1564484This dataset is made available under the Open Database License (http://opendatacommons.org/licenses/odbl/1.0/). The Open Database License (ODbL) is a license agreement intended to allow users to freely share, modify, and use this Dataset while maintaining this same freedom for others, provided that the original source and author(s) are credited. Mateus Oliveira da Cruz, Quimi Vidaurre Montoya, Nicole Marie Gerardo, Andre Rodrigues

Supplementary material 3 Measurements of the microscopic structures of *Manidigitorum* isolates Data type xlsx Explanation note table S8–S12. https://binary.pensoft.net/file/1564485This dataset is made available under the Open Database License (http://opendatacommons.org/licenses/odbl/1.0/). The Open Database License (ODbL) is a license agreement intended to allow users to freely share, modify, and use this Dataset while maintaining this same freedom for others, provided that the original source and author(s) are credited. Mateus Oliveira da Cruz, Quimi Vidaurre Montoya, Nicole Marie Gerardo, Andre Rodrigues
