# Supplementary material for: More diverse than previously thought: a novel Hypocreaceae symbiont from Apterostigma fungus-farming ants
Source: IMA Fungus. 2026 Mar 18;17:e182573. doi: 10.3897/imafungus.17.182573 (PMC13019348; doi:10.3897/imafungus.17.182573)
Supplement: Supplementary material 1 — Metadata of strains used in the phylogenetic analyses [file imafungus-17-e182573-s001.docx]

**More diverse than previously thought: a novel *Hypocreaceae* symbiont from *Apterostigma* fungus-farming ants**

Mateus Oliveira da Cruz, Quimi Vidaurre Montoya, Nicole Marie Gerardo, Andre Rodrigues

Table S1. Metadata of strains used in the phylogenetic analyses.

| **Fungal species name** | **Strain ID** | **City, State, Country** | **Geographical coordinates** | **Habitat** | **Ant colony ID** | **GenBank accessions** | | | | | **References** |
| --- | --- | --- | --- | --- | --- | --- | --- | --- | --- | --- | --- |
|  |  |  |  |  |  | **ITS** | **LSU** | ***tef*1** | ***rpb*1** | ***rpb*2** |  |
| *Escovopsioides nivea* | CBS 135749^ET^ | Viçosa, Minas Gerais, Brazil | 20°44'31.71''S; 42°52 '43.83''W | Fungus garden of *Acromyrmex subterraneus subterraneus* |  | JQ815078 | JQ855716 | JQ855713 | MT305414^#^ | MT305539^#^ | Augustin et al. (2013) |
| *Escovopsioides nivea* | LESF 587 | Camacan, Bahia, Brazil | 15°23'18.2"S; 39°33'30.5"W | Fungus garden of *Atta cephalotes* | BMSR120704-01 | MF116016 | MF116036 | MF140951 | MT305477^#^ | MT305602^#^ | Osti and Rodrigues (2018) |
| *Escovopsioides nivea* | LESF 588 | Camacan, Bahia, Brazil | 15°25'32.3"S 39°32'48.1"W | Fungus garden of *Atta cephalotes* | BMSR120804-01(CA8) | MF116017 | MF116037 | MF140952 | MT305478^#^ | MT305603^#^ | Osti and Rodrigues (2018) |
| *Escovopsioides nivea* | LESF 589 | Camacan, Bahia, Brazil | 15°23'14.8"S; 39°33'28.4"W | Fungus garden of *Atta cephalotes* | BMSR120702-01(FLA4) | MF116018 | MF116038 | MF140953 | MT305479^#^ | MT305604^#^ | Osti and Rodrigues (2018) |
| *Escovopsioides nivea* | LESF 596 | Chuvisca, Rio Grande do Sul, Brazil | 30°50'10.2"S; 51°55'10.4"W | Fungus garden of *Acromyrmex* sp. | AOMB110904-02 | MF116025 | MF116045 | MF140960 | MT305483 | MT305608 | Osti and Rodrigues (2018) |
| *Escovopsioides nivea* | LESF 599 | Sentinela do Sul, Rio Grande do Sul, Brazil |  | Fungus garden of *Acromyrmex heyeri* | AOMB100904-03 | MF116028 | MF116048 | MF140963 | MT305486 | MT305611 | Osti and Rodrigues (2018) |
| *Escovopsioides nivea* | LESF 601 | Rio Claro, São Paulo, Brazil |  | Fungus garden of *Trachymyrmex* sp. | SES080402-04 | MF116029 | MF116049 | MF140964 | MT305487 | MT305612 | Osti and Rodrigues (2018) |
| *Escovopsioides nivea* | LESF 1009 | Manaus, Amazonas, Brazil | 2°26'55.3''S; 59°46'10.9''W | Fungus garden of *Apterostigma* sp. | CALD170307-04 | MT273483 | MT273572 | MT305392 | MT305516 | MT305641 | Montoya et al. (2021) |
| *Escovopsis aspergilloides* | CBS 423.93^ET^ | Trinidad and Tobago, Trinidad |  | Fungus garden of *Trachymyrmex ruthae* |  | NR_137160 | KF293283 | AY172632 | MT305421 | MT305546 | Augustin et al. (2013); Currie et al. (2003); Montoya et al. (2021) |
| *Escovopsis breviramosa* | LESF 055 | Camacan, Bahia, Brazil | 15°23’43.0’’S 39°33’49.1’’W | Fungus garden of *Acromyrmex* sp. | AR110515-01 | KM817044 | OQ589727 | KM817114 | OQ596350 | OQ603820 | Meirelles et al. (2015b); Montoya et al. (2023) |
| *Escovopsis clavata* | CBS 145326^ET^ | Florianópolis, Santa Catarina, Brazil | 27°44’39.6’’S 48°31’10.14’’W | Fungus garden of *Apterostigma* sp. | AR150817-07 | MH715096 | MH724270 | MH715110 | MT305419 | MT305544 | Montoya et al. (2019,  2021) |
| *Escovopsis* *chlamydosporosa* | CBS 149748^ET^ | Novo Airão, Amazonas, Brazil | 2°31’25.6”S 60°49’32.4”W | Fungus garden of *Trachymyrmex* sp. sensu lato | EM170120-01 | OQ589809 | OQ589759 | OQ603902 | OQ596382 | OQ603852 | Montoya et al. (2023) |
| *Escovopsis* *diminuta* | CBS 149747^ET^ | Novo Airão, Amazonas, Brazil | 2°31’23.4’’S 60°49’31.9’’W | Fungus garden of *Trachymyrmex* sp. sensu lato | CAR170120-04 | MT273476 | MT273565 | MT305385 | MT305509 | MT305634 | Montoya et al. (2021) |
| *Escovopsis* *elongatistipitata* | CBS 149750^ET^ | Novo Airão, Amazonas, Brazil | 2°31’23.4’’S 60°49’31.9’’W | Fungus garden of *Trachymyrmex* sp. sensu lato | WM170120-03 | OQ589831 | OQ589781 | OQ603924 | OQ596404 | OQ603874 | Montoya et al. (2023) |
| *Escovopsis* *gracilis* | CBS 149743^ET^ | Camacan, Bahia, Brazil | 14°47’56.8’’S 39°10’16.4’’W | Fungus garden of *Atta cephalotes* | BMSR120703-01 | KM817049 | MH715127 | KM817119 | MT305467 | MT305592 | Meirelles et al. (2015b); Montoya et al. (2023) |
| *Escovopsis* *lentecrescens* | CBS 135750^ET^ | Viçosa, Minas Gerais, Brazil |  | Fungus garden of *Acromyrmex subterraneus molestans* |  | JQ815079 | JQ855717 | JQ855714 | MT305415 | MT305540 | Augustin et al.  (2013); Montoya et  al. (2021) |
| *Escovopsis* *maculosa* | CBS 149746^ET^ | Novo Airão, Amazonas,  Brazil | 2°16’15.7’’S 61°01’8.5’’W | Fungus garden of *Acromyrmex* sp. | AR170124-01 | MT273475 | MT273564 | MT305384 | MT305508 | MT305633 | Montoya et al. (2021) |
| *Escovopsis* *moelleri* | LESF 320 | Viçosa, Minas Gerais, Brazil | 20°44’31.71’’S  42°52’43.83’’W | Fungus garden of *Acromyrmex subterraneus molestans* |  | JQ815077 | JQ855715 | JQ855712 | MT305413 | MT305538 | Augustin et al.  (2013); Meirelles et  al. (2015a); Montoya  et al. (2021) |
| *Escovopsis* *multiformis* | CBS 145327^ET^ | Florianópolis, Santa Catarina, Brazil | 27°28’11.28’’S  48°22’39.48’’W | Fungus garden of *Apterostigma* sp | AR 150816-06 | MH715091 | MH715105 | MH724265 | MT305420 | MT305545 | Montoya et al. (2019,  2021) |
| *Escovopsis* *papillata* | CBS 149745^ET^ | Novo Airão, Amazonas, Brazil | 2°31’25.8’’S 60°49’28.62’’W | Fungus garden of *Apterostigma* sp. | AR170123-01 | OQ589840 | OQ589790 | OQ603933 | OQ596413 | OQ603883 | Montoya et al. (2023) |
| *Escovopsis* *peniculiformis* | CBS 149744^ET^ | Gamboa, Panama |  | Fungus garden of *Atta colombica* | NMG010319-22 | KM817101 | OQ589724 | KM817162 | OQ596347 | OQ603817 | Meirelles et al. (2015b); Montoya et al. (2023) |
| *Escovopsis* *phialicopiosa* | CBS 149738^ET^ | Uberlândia, Minas Gerais, Brazil | 19°17’17.5”S 48°39’40.2”W | *Trachymyrmex* sp. sensu lato | SES080922-03 | KM817088 | OQ589739 | KF240731 | OQ596362 | OQ603832 | Meirelles et al. (2015b); Montoya et al. (2023) |
| *Escovopsis* *pseudocylindrica* | CBS 149749^ET^ | Novo Airão, Amazonas, Brazil | 2°31’29.64’’S 60°49’28.92’’W | Fungus garden of *Trachymyrmex* sp. sensu lato | NMG170120-07 | OQ589819 | OQ589769 | OQ603912 | OQ596392 | OQ603862 | Montoya et al. (2023) |
| *Escovopsis* *rectangula* | CBS 149739^ET^ | Rondonia, Brazil |  | Fungus garden of *Acromyrmex* sp. | SES081007-01 | KM817091 | OQ589729 | KM817152 | OQ596352 | OQ603822 | Meirelles et al. (2015b); Montoya et al. (2023) |
| *Escovopsis* *rosisimilis* | CBS 149742T^ET^ | Uberlândia, Minas  Gerais, Brazil | 19°17’17.5”S 48°39’40.2”W | Fungus garden of *Trachymyrmex*  sp. sensu lato | CTL080820-02 | KM817086 | OQ589740 | KM817148 | OQ596363 | OQ603833 | Meirelles et al. (2015b); Montoya et al. (2023) |
| *Escovopsis* *spicaticlavata* | CBS 149740^ET^ | Manaus, Amazonas,  Brazil | 2°26’54.84’’S 59°46’10.02’’W | Fungus garden of  *Paratrachymyrmex diversus* | SES090109-04 | KM817093 | MH715124 | KM817154 | MT305437 | MT305562 | Meirelles et al.  (2015b); Montoya et  al. (2019) |
| *Escovopsis weberi* | LESF 146 | Corumbataí, São Paulo, Brazil | 22°17'21.7''S; 47°39'22.8''W | Fungus garden of *Atta sexdens rubropilosa* |  | MT273429 | MT273515 | MT305344 | MT305441 | MT305566 | Montoya et al. (2021) |
| *Hypomyces asterophorum* | TFC 201316 | Madagascar |  | *Eucalyptus* forest |  | FN859414 | FN859414 | FN868732 | FN868795 | FN868668 | Põldmaa (2011) |
| *Hypomyces asterophorum* | CBS 67677^ET^ | Japan |  |  |  | FN859395 | AJ583469 | FN868712 | FN868776 | FN868649 | Põldmaa (2011) |
| *Hypomyces samuelsii* | TFC 2007-23 | Peru |  | on  basidioma of an agaricoid basidiomycete on a stem of a palm |  | FN859451 | FN859451 | FN868769 | FN868828 | FN868705 | Põldmaa (2011) |
| *Hypomyces semicirculare* | CBS 705. 88^ET^ | Cuba |  | On old  polypore |  | NR_121425 | FN859417 | FN868735 | FN868671 | FN868798 | Põldmaa (2011) |
| *Lecanicillium antillanum* | CBS 350.85^ET^ | Cuba |  | on  basidioma of an agaricoid |  | NR_111097 | AF339536 | DQ522350 | DQ522396 | DQ522450 | Spatafora et al. (2007) |
| *Luteomyces trichodermoides* | CBS137343^ET^ | Rio Claro, São Paulo, Brazil |  | Fungus garden of *Mycocepurus goeldi* |  | NR_158383 | MT305542 | KF033128 | MT305417 | MT305542.1 | Masiulionis et al. (2015); Montoya et al. (2021) |
| *Luteomyces trichodermoides* | LESF 310 | Florianópolis, Santa Catarina, Brazil | 27°37'49.62''S, 48°27'3.6''W | Fungus garden of *Mycetophylax morschi* | AR140226-04 | MH715088 | MH715102 | MH724262 | MT305460 | MT305585 | Montoya et al. (2019) |
| *Luteomyces trichodermoides* | LESF 311 | Florianópolis, Santa Catarina, Brazil | 27°37'49.62''S, 48°27'3.6''W | Fungus garden of *Mycetophylax morschi* | AR140226-04 | MH715089 | MH715103 | MH724263 | MT305461 | MT305586 | Montoya et al. (2019) |
| *Luteomyces trichodermoides* | LESF 312 | Florianópolis, Santa Catarina, Brazil | 27°37'49.62''S, 48°27'3.6''W | Fungus garden of *Mycetophylax morschi* | AR140226-04 | MH715090 | MH715104 | MH724264 | MT305462 | MT305587 | Montoya et al. (2019) |
| *Luteomyces trichodermoides* | LESF 832 | Rio Claro, São Paulo, Brazil |  | Fungus garden of *Mycocepurus smithii* | CR141003-02 | MT273453 | MT273542 | MT305366 | MT305488 | MT305613 | Montoya et al. (2021) |
| *Luteomyces trichodermoides* | LESF 835 | Rio Claro, São Paulo, Brazil |  | Midden of *Mycocepurus smithii* | CR141010 | MT273456 | MT273545 | MT305369 | MT305491 | MT305616 | Montoya et al. (2021) |
| *Luteomyces trichodermoides* | LESF 833 | Rio Claro, São Paulo, Brazil |  | Fungus garden of *Mycocepurus smithii* | CR141003-02 | MT273454 | MT273543 | MT305367 | MT305489 | MT305614 | Montoya et al. (2021) |
| *Luteomyces trichodermoides* | LESF 834 | Rio Claro, São Paulo, Brazil |  | Fungus garden of *Mycocepurus smithii* | CR141003-02 | MT273455 | MT273544 | MT305368 | MT305490 | MT305615 | Montoya et al. (2021) |
| *Manidigitorum attinorum* | CBS 153793^ET^ | Belterra, Pará, Brazil | 3°02'54.9"S 54°55'41.0"W | *Apterostigma urichii* | CALD170314-03 | PX225019 | PX225029 | PX633031 | PX663411 | PX663422 | This study |
| *Manidigitorum attinorum* | LESF 2006 | Parauapebas, Pará, Brazil | 6°07'57.5" S 50°21'33.7" W | *Apterostigma pilosum* group | QVM241008-02 | PX225024 | PX225030 | PX633032 | PX663412 | PX663423 | This study. |
| *Manidigitorum cervicornutus* | CBS 153791^ET^ | Rancho Frio, Darién Province, Panamá | 8°01'11.0"S 77°43'56.5"W | *Apterostigma pilosum* group | UGM030106-02 | PX225025 | PX225026 | PX633029 | PX663406 | PX663417 | This study. |
| *Manidigitorum cervicornutus* | LESF 889 | Colón Province, Panamá |  | *Apterostigma dentigerum* | NMG020521-04 | PX225016 | PX225027 | PX633031 | PX663407 | PX663418 | This study. |
| *Manidigitorum cervicornutus* | LESF 890 | Panamá |  | *Apterostigma pilosum* group |  | PX225018 | PX225028 | PX633035 | PX663408 | PX663419 | This study. |
| *Manidigitorum minutus* | CBS 153792^ET^ | Novo Airão, Amazonas, Brazil | 2° 31' 23,4 S 60° 49' 31,9 W | *Apterostigma urichii* | CAR170120-04 | PX225022 | PX225031 | PX633038 | PX663415 | PX663425 | This study. |
| *Manidigitorum minutus* | LESF 1036 | Novo Airão, Amazonas, Brazil |  | *Apterostigma pilosum* group | CC170119-03 | PX225023 | PX225032 | PX633039 | PX663416 | PX663426 | This study. |
| *Manidigitorum ramosus* | LESF 1934 | Ribeirão Grande, São Paulo, Brazil | 24°16'44.9"S 48°24'46.7"W | *Apterostigma pilosum* group | DSA240510-01 | PX225015 | PX225033 | PX633034 | PX663413 | PX663427 | This study. |
| *Manidigitorum ramosus* | CBS 153795^ET^ | Tiputini Biodiversity Station, Ecuador |  | *Apterostigma* cf. *dentigerum* | AGH030609-03 | PX225020 | PX225034 | PX633033 | PX663414 | PX663424 | This study. |
| *Manidigitorum sessilis* | LESF 875 | Bocas del Toro Province, Panamá |  | *Apterostigma* cf. *dentigerum* | UGM020602-07 | PX225017 | PX225035 | PX633036 | PX663409 | PX663420 | This study. |
| *Manidigitorum sessilis* | CBS 153794^ET^ | Bocas del Toro Province, Panamá |  | *Apterostigma pilosum* group |  | PX225021 | PX225036 | PX633037 | PX663410 | PX663421 | This study. |
| *Protocrea pallida* | TFC 99209^ET^ | New York, Cleaveland |  |  |  | NR_111329 | EU710769 | EU703903 |  | EU703949 | Jaklitsch (2011) |
| *Sphaerostilbella aureonitens* | GJS 74-87 |  |  |  |  | FJ442633 | HM466683 | FJ467644 |  | FJ442763 | unpublished |
| *Sympodiorosea kreiselii* | LESF 053 | Florianópolis, Santa Catarina, Brazil | 27°37'50.01''S 48°27'03.64''W | Fungus garden of *Mycetophylax morschi* | AR090306-01 | KJ808767 | KJ808765 | KJ 808766 | MT305418 | MT305543 | Meirelles et al. (2015a) |
| *Sympodiorosea kreiselii* | LESF 302 | Florianópolis, Santa Catarina, Brazil | 27°31'24.96''S  48°25'3.78''W | Fungus garden of *Mycetophylax morschi* | AR140227-05 | MH715085 | MH715099 | MH724259 | MT305452 | MT305577 | Montoya et al. (2019) |
| *Sympodiorosea kreiselii* | LESF 303 | Florianópolis, Santa Catarina, Brazil | 27°31'24.96''S  48°25'3.78''W | Fungus garden of *Mycetophylax morschi* | AR140227-05 | MH715086 | MH715100 | MH724260 | MT305453 | MT305578 | Montoya et al. (2019) |
| *Sympodiorosea kreiselii* | LESF 304 | Florianópolis, Santa Catarina, Brazil | 27°31'24.96''S  48°25'3.78''W | Fungus garden of *Mycetophylax morschi* | AR140227-05 | MH715087 | MH715101 | MH724261 | MT305454 | MT305579 | Montoya et al. (2019) |
| *Sympodiorosea kreiselii* | LESF 305 | Florianópolis, Santa Catarina, Brazil | 27°37'49.6"S; 48°27'03.6"W | Fungus garden of *Mycetophylax morschi* | AR140226-01 | MT273438 | MT273524 | MT305353 | MT305455 | MT305580 | Montoya et al. (2021) |
| *Sympodiorosea kreiselii* | LESF 306 | Florianópolis, Santa Catarina, Brazil | 27°31'25.0"S; 48°25'03.8"W | Fungus garden of *Mycetophylax morschi* | AR140227-05 | MT273439 | MT273525 | MT305354 | MT305456 | MT305581 | Montoya et al. (2021) |
| *Sympodiorosea kreiselii* | LESF 307 | Florianópolis, Santa Catarina, Brazil | 27°31'25.0"S; 48°25'03.8"W | Fungus garden of *Mycetophylax morschi* | AR140227-05 | MT273440 | MT273526 | MT305355 | MT305457 | MT305582 | Montoya et al. (2021) |
| *Sympodiorosea kreiselii* | LESF 308 | Florianópolis, Santa Catarina, Brazil | 27°37'49.6"S; 48°27'03.6"W | Fungus garden of *Mycetophylax morschi* | AR140226-04 | MT273441 | MT273527 | MT305356 | MT305458 | MT305583 | Montoya et al. (2021) |
| *Sympodiorosea kreiselii* | LESF 309 | Florianópolis, Santa Catarina, Brazil | 27°37'47.9"S; 48°27'04.0"W | Fungus garden of *Mycetophylax morschi* | AR140226-05 | MT273442 | MT273528 | MT305357 | MT305459 | MT305584^#^ | Montoya et al. (2021) |
| *Sympodiorosea* sp. | LESF 864 | Iguazu, Argentina |  | *Mycetarotes parallelus* | SES030331-05 | MT273464 | MT273553 | MT305375 | MT305499 | MT305624 | Montoya et al. (2021) |
| *Sympodiorosea* sp. | LESF 886 |  |  |  |  | MT273468 | MT273557 | MT305378 | MT305502 | MT305627 | Montoya et al. (2021) |
| *Sympodiorosea* sp. | LESF 887 |  |  |  |  | MT273469 | MT273558 | MT305379 | MT305503 | MT305628 | Montoya et al. (2021) |
| *Sympodiorosea* sp. | LESF 899 | Botucatu, São Paulo, Brazil | 22°54'19.6''S; 48°14'33.7''W | Fungus garden of *Mycocepurus goeldii* | QVM160527-03 | MT273473 | MT273562 | MT305382 | MT305506 | MT305631 | Montoya et al. (2021) |
| *Sympodiorosea* sp. | LESF 1010 | Manaus, Amazonas, Brazil | 2°26'51.6''S; 59°45'53.4''W | Fungus garden of *Apterostigma* sp. | CALD170309-02 | MT273484 | MT273573 | MT305393 | MT305517 | MT3 05642 | Montoya et al. (2021) |
| *Trichoderma harzianum* | CBS 22695 | England |  |  |  | AY605713 | HM466680 | AF534621 | JQ031082 | AF545549 | Chaverri et al. (2003) |

^ET^ ex-type cultures.

**Reference****s**

Augustin JO, Groenewald JZ, Nascimento RJ, Mizubuti ESG, Barreto RW, Elliot SL, Evans HC (2013). Yet more “weeds” in the garden: Fungal novelties from nests of leaf-cutting ants. PLoS One 8(12): e82265. <https://doi.org/10.1371/journal.pone.0082265>

Chaverri P, Castlebury LA, Samuels GJ, Geiser DM (2003). Multilocus phylogenetic structure within the *Trichoderma harzianum* / *Hypocrea lixii* complex. Molecular Phylogenetic and Evolution 27: 302–313. <https://doi.org/10.1016/S1055-7903(02)00400-1>.

Currie CR, Wong B, Stuart AE, Schultz TR, Rehner SA, Mueller UG, Sung GH, Spatafora JW, Straus NA (2003) Ancient tripartite coevolution in the attine ant–microbe symbiosis. Science 299: 386–388. <https://doi.org/10.1126/science.1078155>.

Jaklitsch WM (2011) European species of *Hypocrea* part II: species with hyaline ascospores. Fungal Diversty 48: 1-250. https//doi: 10.1007/s13225-011-0088-y.

Masiulionis VE, Cabello MN, Seifert KA, Rodrigues A, Pagnocca FC (2015). *Escovopsis trichodermoides* sp. nov., isolated from a fungus garden of the lower attine ant *Mycocepurus goeldii*. Antonie van Leeuwenhoek 107: 31–40. <https://doi.org/10.1007/s10482-014-0367-1>.

Meirelles LA, Montoya QV, Solomon SE, Rodrigues A (2015a) New light on the systematics of fungi associated with attine ant gardens and the description of *Escovopsis* *kreiselii* sp. nov. PLoS One 10: e0112067. <https://doi.org./10.1371/journal.pone.0112067>.

Meirelles LA, Solomon SE, Bacci M, Wright AM, Mueller UG, Rodrigues A (2015b) Shared *Escovopsis* parasites between leaf-cutting and non-leaf-cutting ants in the higher attine fungus-growing ant symbiosis. Royal Society Open Science 2: e150257. <https://doi.org/10.1098/rsos.150257>.

Montoya QV, Martiarena MJS, Polezel DA, Kakazu S, Rodrigues A (2019) More pieces to a huge puzzle: Two new *Escovopsis* species from fungus gardens of attine ants. MycoKeys 46: 97–118. <https://doi.org/10.3897/mycokeys.46.30951>.

Montoya QV, Martiarena MJS, Bizarria R, Gerardo NM, Rodrigues A (2021) Fungi inhabiting attine ant colonies: Reassessment of the genus *Escovopsis* and description of *Luteomyces* and *Sympodiorosea* gens. nov. IMA Fungus 12: 23. <https://doi.org/10.1186/s43008-021-00078-8>

Montoya QV, Martiarena MJS, Rodrigues A (2023) Taxonomy and systematics of the fungus-growing ant associate *Escovopsis* (*Hypocreaceae*). Studies in Mycology 106: 349–397. <https://doi.org/10.3114/sim.2023.106.06>.

Osti JF, Rodrigues A (2018). *Escovopsioides* as a fungal antagonist of the fungus cultivated by leafcutter ants. BMC Microbiology 18: 130.

<https://doi.org/10.1186/s12866-018-1265-x>.

Põldmaa K (2011) Tropical species of *Cladobotryum* and *Hypomyces* producing red pigments. Studies in Mycology 68: 1–34. <https://doi.org/10.3114/sim.2011.68.01>

Spatafora JW, Sung GH, Sung JM, Hywel-Jones NL, White JF Jr (2007). Phylogenetic evidence for an animal pathogen origin of ergot and the grass endophytes. Molecular Ecology 16: 1701–1711. <https://doi.org/10.1111/j.1365-294X.2007.03225.x>.
